# p53-dependent programmed necrosis controls germ cell homeostasis during spermatogenesis

**DOI:** 10.1371/journal.pgen.1007024

**Published:** 2017-09-25

**Authors:** Francesco Napoletano, Benjamin Gibert, Keren Yacobi-Sharon, Stéphane Vincent, Clémentine Favrot, Patrick Mehlen, Victor Girard, Margaux Teil, Gilles Chatelain, Ludivine Walter, Eli Arama, Bertrand Mollereau

**Affiliations:** 1 Laboratory of Biology and Modelling of the Cell, UMR5239 CNRS/Ecole Normale Supérieure de Lyon, INSERM U1210, UMS 3444 Biosciences Lyon Gerland, Université de Lyon, Lyon, France; 2 Apoptosis, Cancer and Development Laboratory- Equipe labellisée ‘La Ligue’, LabEx DEVweCAN, Centre de Cancérologie de Lyon, INSERM U1052-CNRS UMR5286, Université de Lyon, Centre Léon Bérard, Lyon, France; 3 Department of Molecular Genetics, Weizmann Institute of Science, Rehovot, Israel; The University of North Carolina at Chapel Hill, UNITED STATES

## Abstract

The importance of regulated necrosis in pathologies such as cerebral stroke and myocardial infarction is now fully recognized. However, the physiological relevance of regulated necrosis remains unclear. Here, we report a conserved role for p53 in regulating necrosis in *Drosophila* and mammalian spermatogenesis. We found that *Drosophila* p53 is required for the programmed necrosis that occurs spontaneously in mitotic germ cells during spermatogenesis. This form of necrosis involved an atypical function of the initiator caspase Dronc/Caspase 9, independent of its catalytic activity. Prevention of p53-dependent necrosis resulted in testicular hyperplasia, which was reversed by restoring necrosis in spermatogonia. In mouse testes, p53 was required for heat-induced germ cell necrosis, indicating that regulation of necrosis is a primordial function of *p53* conserved from invertebrates to vertebrates. *Drosophila* and mouse spermatogenesis will thus be useful models to identify inducers of necrosis to treat cancers that are refractory to apoptosis.

## Author summary

Cell death allows elimination of supernumerary cells during development or of abnormal cells throughout life. Physiological cell death is tightly regulated to prevent pathologies such as degenerative diseases or cancers, which often occur due to excessive or absent cell death, respectively. Understanding the mechanisms of cell death pathways is thus crucial for fighting various diseases. The best studied form of cell death, apoptosis, has classically been considered the sole form of cell death during development, while other forms of cell death, referred to as necrosis, were considered accidental. Here, we show that a regulated form of necrosis controls germ cell number during *Drosophila* spermatogenesis, thus demonstrating that necrosis can play a key role in controlling cell number in physiological conditions. This regulated form of necrosis involves p53, a protein frequently mutated in cancer. Furthermore, we demonstrate that this pathway prevents tissue hyperplasia, a condition that can lead to cancer. We also provide evidence that the same pathway is operational in mammals, as shown in the testes of mice subjected to mild heat stress. These findings expand the possible mechanisms by which cell death could be targeted to treat human pathologies.

## Introduction

Cell death can occur as part of a physiological program; for example, during development and tissue homeostasis, and can also be induced in pathologies when adaptive cellular responses to adversity fail. Necrosis has long been considered an accidental process, but it has become increasingly clear that it is in fact a regulated form of cell death with great pathological relevance [1, 2, 3, 4]. However, little is known about the involvement of regulated necrosis in programmed cell death, largely because few animal models have been developed for studying physiological necrotic cell death mechanisms. Moreover, only a few markers can be used to detect necrosis *in vivo*, and those rely heavily on ultrastructural analysis. Necrosis is interestingly associated with cellular and organelle swelling, leading to rupture of cellular plasma and nuclear membranes. This contrasts with apoptosis, which involves cellular shrinkage and nuclear condensation but leaves organelles and cellular membranes intact. Since the loss of membrane integrity is a central feature of necrotic cell death, incorporation of the membrane-impermeant dye propidium iodide (PI) has been used to detect necrotic cells *in vivo* [5]. Interestingly, DNA fragmentation, most commonly visualized by the TUNEL assay, cannot discriminate between apoptosis and necrosis unless it is combined with staining of activated effector enzymes known as caspases [6]. Caspases (cysteine-dependent aspartate-directed proteases) are best known as mediators of apoptosis but they also have important non-apoptotic functions [7]. During apoptosis, initiator caspases (e.g., Caspase-8 and -9) cleave and activate effector caspases (e.g., Caspase-3 and -7), which subsequently activate or inactivate additional substrates by cleavage, leading to cell death. The initiator and effector caspases carry long and short prodomains, respectively. The long prodomain Caspase Recruitment Domain (CARD) present in Casp-9 and its *Drosophila* homolog *Dronc* allows protein–protein interactions and Casp-9/Dronc activation *via* the apoptosome, a multimeric structure containing the Apoptotic protease activating factor-1 (Apaf-1). In contrast, effector caspases activation requires proteolytic cleavage by initiator caspases. Recent studies indicate that initiator caspases have functions independent of their catalytic activity and some act as scaffold proteins in signaling for non-apoptotic processes such as inflammation [8] or apoptosis-induced proliferation [9].

p53, the product of the tumor suppressor gene *TP53*, induces canonical cellular responses such as cell cycle arrest, senescence, and apoptosis, which contribute to tumor suppression. In the past few years, there has been considerable interest in the role of non-canonical functions of p53, including the regulation of autophagy, cellular metabolism, stem cell function, and necrosis, and their potential role in tumor suppression [10]. p53 is conserved from invertebrates to mammals, and it has been proposed that its tumor-suppressive activity was co-opted from more ancient functions [11]. Thus, studying p53 in a simple organism such as *Drosophila* will help to identify its functions relevant to tumor suppression [12].

**Fig 1:**
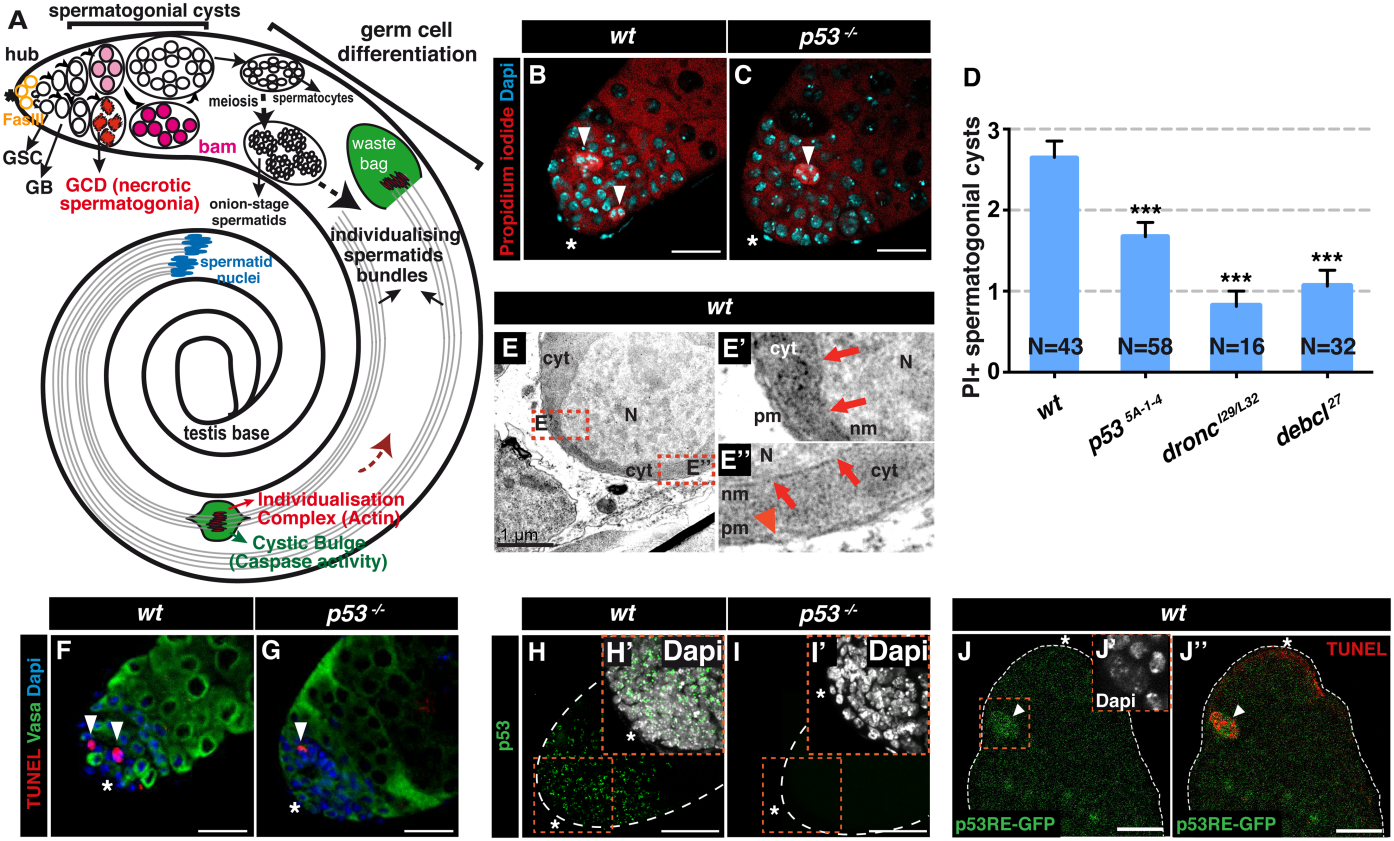
*Drosophila* p53 is required for necrotic cell death during spermatogenesis. (**A**) In *Drosophila* testis apical tip, germ stem cells (GSCs) in contact with somatic hub cells (asterisk), which express Fasciclin III (FasIII), self-renew and generate goniablasts (GB) that produce spermatogonial cysts, some of which are eliminated by necrosis (germ cell death [GCD], in red). Increasing level of Bam (pink to magenta) induce maturation of spermatogonia into spermatocytes, which produce cysts of 64 spermatids by meiosis. Spermatids elongate with nuclei at the base of the testis (blue) and undergo individualization when F-actin investment cones form the individualization complex (IC, red). The IC moves toward the sperm tails (brown dashed arrow) within a structure known as the cystic bulge, which then forms the waste bag. Somatic cells and the seminal vesicle are omitted, and cell size is not to scale. (**B, C**) Propidium iodide (PI) staining of *wt* (**B**) and *p53*^*-/-*^ (*p53*^*5A-1-4*^, **C**) testes. Necrotic cells are indicated with white arrowheads. Nuclei are stained with DAPI. Scale bar, 40 μm. (**D**) Quantification of PI^+^ GCs in *wt* and *p53*^*-/-*^ (*p53*^*5A-1-4*^*)*, *dronc*^*-/-*^ (*dronc*^*I29/L32*^), and *debcl*^*-/-*^ (*debcl*^*27*^) mutant testes (mean ± s.e.m. of three independent experiments, N testes/genotype). ***p < 0.001 by two-tailed unpaired Student’s t-test. (**E-E”**) Electron micrographs of *wt* necrotic GC. Nucleus (N) and cytoplasm (cyt) are indicated. In the magnified views (**E'** and **E”** indicated by dashed red box in **E**) red arrowhead and arrows indicate plasma membrane (pm) and nuclear membrane (nm) ruptures, respectively. Scale bar in **E**, 1 μm. (**F, G**) TUNEL^+^ Vasa^+^ spermatogonial cysts (arrowheads) in *wt* (**F**) and *p53*^*-/-*^ (*p53*^*5A-1-4*^, **G**) adult *Drosophila* testes. TUNEL^+^ cells are indicated with white arrowheads. Nuclei are stained with DAPI and the hub region is indicated with a white asterisk. Scale bar, 40 μm. (**H**, **I**) p53 immunostaining of wild-type (*wt*) and *p53*^*-/-*^ adult *Drosophila* testes. Nuclei are stained with DAPI (insets **H'**, **I'**). Scale bar, 40 μm. (**J-J”**) GFP immunostaining (green in **J** and **J”**) of adult *Drosophila* testes harboring the *p53RE-GFPnls* reporter and co-stained for TUNEL (red in **J”**). A GFP^+^ TUNEL^+^ necrotic spermatogonial cyst (orange box in **J**) is indicated by a white arrowhead (**J** and **J”**). The hub region is indicated with a white asterisk (**J** and **J”**). Nuclei are stained with DAPI (inset **J'** corresponding to the orange box in **J**). Scale bar, 30 μm.

Recent work has shown that during adult *Drosophila* spermatogenesis, 20% to 30% of spermatogonial cysts are normally eliminated by a form of programmed germ cell (GC) death with morphological features reminiscent of necrosis [13]. GC death requires an atypical function of Casp-9/Dronc acting independently of the apoptosome, but the exact nature of the death mechanism remains to be elucidated. By contrast, the canonical apoptosome-dependent function of Dronc/Casp-9 is used for spermatid individualization at a later stage of spermatogenesis [14, 15] (Fig 1A). In mouse spermatogenesis, the first round of GC mitosis is accompanied by a massive and early wave of apoptosis that requires Tp53 [16], which is followed by necrotic GC death at sexual maturity [17]. Although germ cells with necrotic features had previously been observed in adult mice, the potential importance of this form of cell death in mammalian spermatogenesis was not examined at that time [18]. In vertebrates, necrosis is regulated by a number of pathways, among which the best characterized is necroptosis mediated by receptor interacting protein kinase-3 (RIPK3) and its substrate mixed lineage kinase like (MLKL) [19]. Another necrotic pathway involves activation of Tp53 in response to reactive oxygen species, possibly in a RIPK1-independent manner [20, 21, 22, 23]. Since the *Drosophila* genome does not encode a RIPK homolog, the fly provides a genetic model to investigate the mechanisms underlying p53 involvement in necrosis independently of RIPK.

## Results

### p53 mediates programmed necrosis during *Drosophila* spermatogenesis

During *Drosophila* spermatogenesis, male germ stem cells (GSCs), located at the apical tip of the testis in contact with somatic hub cells, divide to form goniablasts that undergo four rounds of mitotic divisions with incomplete cytokinesis. The resulting cysts contain 16 spermatogonia that mature into spermatocytes before entering meiosis [24, 25] (Fig 1A). GC death occurring during spermatogenesis is independent of the apoptosome and has some morphological features of necrosis [13]; therefore, we hypothesized that GC death may involve a physiological necrotic program. We first asked whether GC death culminated in cellular membrane rupture, which is a typical feature of necrosis but is not observed in apoptosis. By monitoring the incorporation of PI, a membrane-impermeable dye used to assess necrosis *in vivo* [5], we detected PI-positive (PI^+^) cells among the proliferating GCs at the apical tip of wild-type (*wt)* adult *Drosophila* testes (Fig 1B), indicating that GCs die by necrosis. To substantiate this observation, we analyzed the integrity of the nuclear and plasma membranes of dying GCs by electron microscopy and observed membrane rupture at several locations (Fig 1E–1E”). Interestingly, GC death was also detected by the TUNEL assay (Fig 1F), as previously reported [13]. This was not unexpected because earlier observations in rat liver showed that TUNEL can label fragmented DNA in both apoptotic and necrotic cells [6]. To assess whether TUNEL labels necrotic GCs, we performed PI/TUNEL double labeling in *wt Drosophila* testes (S1A and S1B”’ Fig). We observed GCs that were positive for both PI and TUNEL (S1A–S1A”’ Fig), confirming that DNA fragmentation occurs during necrosis and that necrotic GCs are labeled by TUNEL. We also found PI^+^ or TUNEL^+^ single-labeled dying GCs (S1B–S1B”’ Fig), suggesting that TUNEL and PI staining can occur sequentially. Next, we examined the expression of the activated apoptosis executioner Dcp-1 (effector-like caspase) in dying TUNEL^+^ GCs. We found that activated Dcp-1 was readily detected in terminally differentiating sperm, as previously described [15], but not in dying GCs (S1C–S1C” Fig), confirming the lack of apoptosis. These results demonstrate that programmed (spontaneous) GC death is accompanied by cellular membrane rupture and the absence of caspase activation, indicating a necrotic rather than apoptotic form of cell death.

We next asked whether GC death requires p53. At the testis apical tip, p53 expression, detected by specific antibody staining, was observed in GSCs and spermatogonia but was largely absent from somatic hub cells and spermatocytes ([26] and Fig 1H–1I’). PI and TUNEL staining revealed fewer PI^+^ or TUNEL^+^ spermatogonial cysts in the testes of *p53*^*-/-*^ null flies (*p53*^*5A-1-4*^ and *p53*^*E8*^) compared with *wt* flies (Fig 1C, 1D, 1G and S1D Fig), indicating a role for p53 in GC death. It was previously shown with TUNEL detection that two genes required for GC death, *Debcl* (fly homolog of *Bax*) and *Dronc/Casp-9*, exhibit fewer TUNEL^+^ GCs compared with *wt* [13]. We found that *Debcl* and *Dronc/Casp-9* mutant flies not only showed drastically fewer TUNEL^+^ but also PI^+^ GC (Fig 1D and S1D Fig), suggesting that *Debcl* and *Dronc/Casp-9* are acting together with p53 in the execution of necrosis. Although p53 has been proposed to display non-nuclear functions in some contexts [27], it is best characterized as a transcription factor. To investigate how p53 mediates GC death in spermatogonia, we monitored a nuclear GFP reporter protein expressed in the nucleus under the control of the p53 responsive element (*p53RE-GFPnls*) [28] in the testes of *wt* flies. GFP expression was induced selectively in dying spermatogonia (Fig 1J–1J”), indicating that GC death was associated with the transcriptional activity of p53. The *Drosophila p53* locus encodes three isoforms: full-length Dp53/p53B, N-terminally truncated DΔNp53/p53A (corresponding to the human full-length TAp53 and N-terminally truncated TAΔNp53 forms, respectively), and p53E [29, 30, 31]. Interestingly, spermatogonia-specific silencing (with the *nanos* [*nos*] driver) of the Dp53/TAp53 isoform in *wt* flies reduced the level of GC death to that observed in *p53*^*-/-*^ flies (S1E Fig), suggesting a major role for Dp53/TAp53 in male GC homeostasis. Collectively, these data demonstrate that p53 contributes to physiological GC death in the *Drosophila* testis, likely via its transcriptional function, and show that p53-dependent GC death exhibits hallmarks of necrosis.

**Fig 2:**
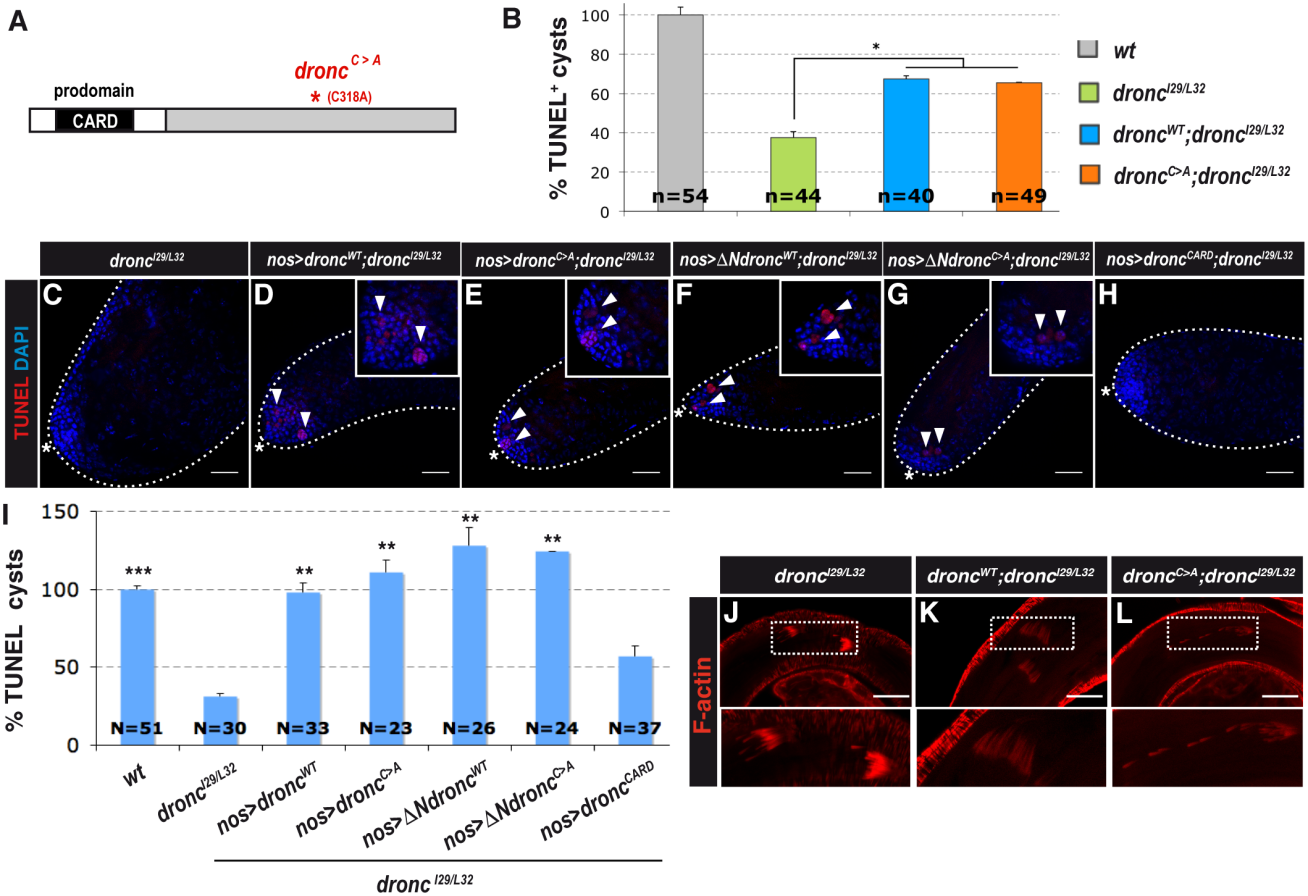
Atypical Dronc function is required for necrotic cell death. **(A)** Schematic of Dronc protein highlighting the N-terminal containing CARD domain and the Dronc^C>A^ mutation in the catalytic domain. (**B**) Quantification of TUNEL^+^ spermatogonial cysts of adult testes from *dronc*^*I29/L32*^ mutant flies expressing wild-type (*dronc*^*WT*^) or catalytically inactive (*dronc*^*C>A*^) *dronc* under the control of the endogenous promoter sequences. Quantification is expressed as % of *wt* (mean ± s.e.m. of three independent experiments, N testes/genotype). *p < 0.05 by two-tailed unpaired Student’s t-test. (**C**-**H**) TUNEL staining of spermatogonial cysts (white arrowheads in **D**-**G**) in adult testes from *dronc*^*I29/L32*^ mutant flies (**C**) expressing full-length *dronc*^*WT*^ (**D**), full-length *dronc*^*C>A*^ (**E**), CARD prodomain-deleted wild-type *dronc* (*ΔNdronc*^*WT*^, **F**), CARD prodomain-deleted catalytically inactive *dronc* (*ΔNdronc*^*C>A*^, **G**), or the CARD prodomain only (*dronc*^*CARD*^, **H**) under the control of the *nos* driver. Nuclei are stained with DAPI and the hub region is indicated by the white asterisk. Scale bar, 40 μm. (**I**) Quantification of TUNEL^+^ spermatogonial cysts expressed as % of *wt* (mean ± s.e.m. of three independent experiments, N testes/genotype). **p < 0.01, ***p < 0.001 versus *dronc*^*I29/L32*^ by two-tailed unpaired Student’s t-test. (**J**-**L**) Phalloidin staining of F-actin-rich investment cones (dotted rectangles, enlarged in insets) in adult testes from *dronc*^*I29/L32*^ mutant flies (**J**), expressing wild-type *dronc* (*dronc*^*WT*^, **K**) or catalytically inactive *dronc* (*dronc*^*C>A*^, **L**) under the control of endogenous promoter sequences. Scale bar, 40 μm.

Previous work has shown that the role of Dronc/Casp-9 in GC death occurs independently of the apoptosome [13]. To investigate the apparently paradoxical role of this pro-apoptotic caspase in necrotic GC death, we analyzed flies carrying a catalytically dead form of Dronc/Casp-9 (Dronc^C>A^) to abolish the apoptotic function of Dronc/Casp-9 and performed rescue experiments with Dronc^WT^ and Dronc^C>A^ (Fig 2A). Notably, re-expression of either *Dronc*^*WT*^ or *Dronc*^*C>A*^ under the control of *Dronc* endogenous promoter sequences was sufficient to restore GC death in *Dronc*/*Casp-9* mutant testes (Fig 2B). We also examined the importance of the Dronc/Casp-9 N-terminal prodomain (containing a CARD, Fig 2A), which is required for Dronc/Casp-9 interaction with Apaf-1 in the apoptosome [32]. For this, CARD-deleted Dronc/Casp-9 variants (*ΔNdronc or ΔNdronc*^*C>A*^) were expressed specifically in the spermatogonia of *Dronc/Casp-9* mutants using the *nos* driver [33]. We found that expression of *ΔNdronc* or *ΔNdronc*^*C>A*^ but not of the CARD domain alone (*dronc*^*CARD*^) restored GC death in *Dronc/Casp-9* mutants (Fig 2C–2I). This demonstrates that Dronc/Casp-9 does not require its catalytic activity or the CARD domain to regulate GC death and that Dronc acts independently of Dark (the fly homolog of Apaf-1) for the induction of necrosis. The latter finding is in agreement with the observation that loss of Dark/Apaf-1 function does not suppress GC death [13]. In contrast to the role of Dronc/Casp-9 in GC death, its catalytic activity is required to rescue the caspase-dependent coordinated movement of investment cones during sperm differentiation (Fig 2J–2L). Thus, these data demonstrate that Dp53/Tp53 and an atypical catalysis-independent function of Dronc/Casp-9 regulate programmed necrosis during spermatogenesis, whereas catalytically active Dronc/Casp-9 and the apoptosome regulate sperm individualization.

### p53-dependent necrosis suppresses hyperplasia during *Drosophila* spermatogenesis

**Fig 3:**
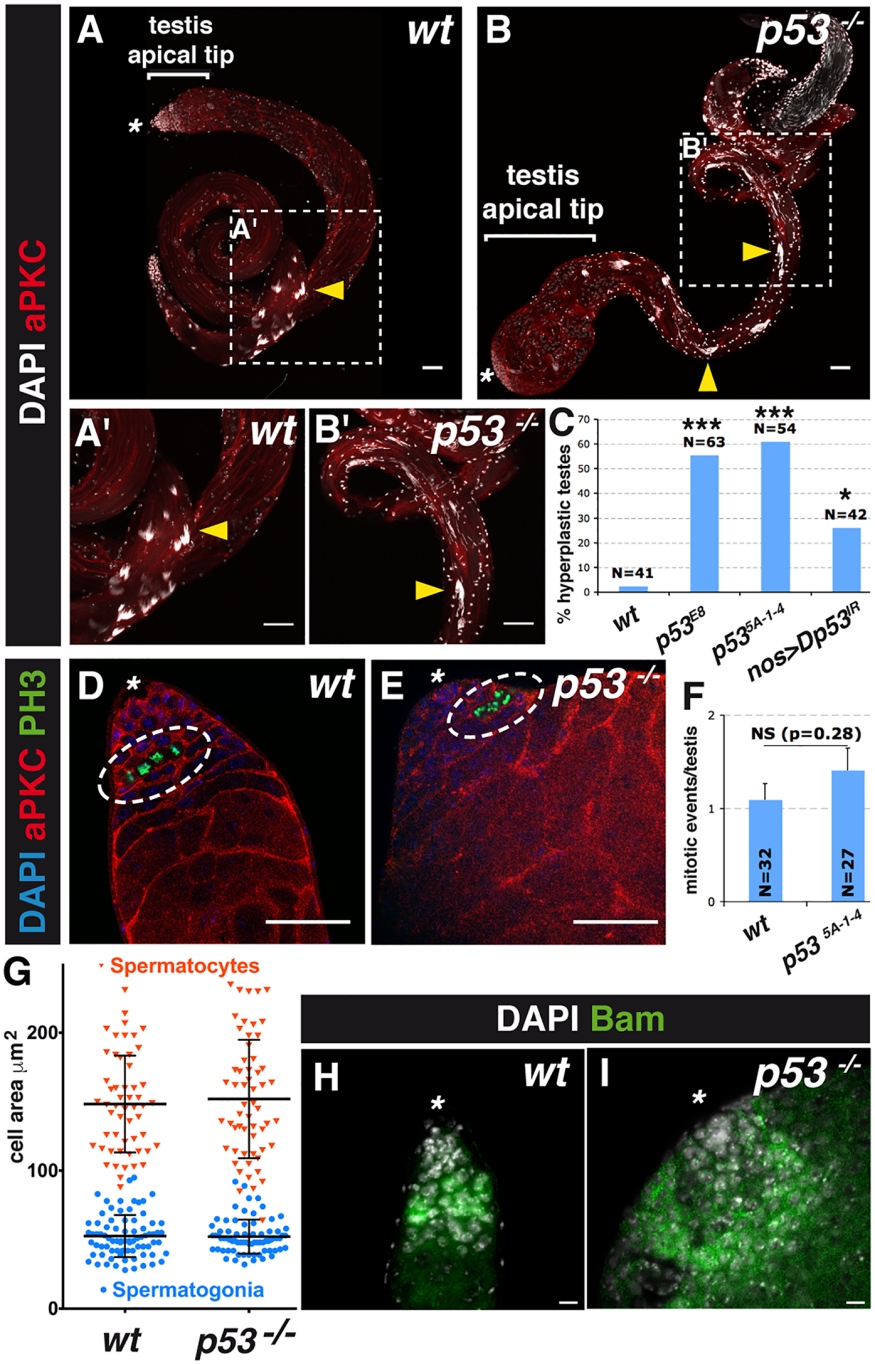
p53 suppresses testicular hyperplasia in *Drosophila*. (**A**-**B'**) aPKC immunostaining of wild-type (*wt*, **A**, **A'**) and *p53*^*-/-*^ (*p53*^*5A-1-4*^, **B**, **B'**) adult *Drosophila* testes. The testis apical tip and hub region are indicated by the white bar and asterisk, respectively. Nuclei are stained with DAPI. Arrowheads indicate spermatid nuclei. Scale bar, 40 μm. (**C**) Frequency of adult testes (*wt* and *p53*^*E8*^ 4 days post-eclosion, *p53*^*5A-1-4*^ 18 days post-eclosion, *nos>Dp53*^*IR*^ 10 days post-eclosion) with apical tip hyperplasia (mean ± s.e.m. of three independent experiments, N testes/genotype). *p < 0.05, ***p < 0.001 versus *wt* flies by Fisher’s exact test. (**D**, **E**) PH3 and aPKC double immunostaining of *wt* (**D**) and *p53*^*-/-*^ (*p53*^*E8*^, **E**) adult *Drosophila* testes. White dotted ovals mark PH3^+^ spermatogonial cysts. Nuclei are stained with DAPI. Scale bar, 40 μm. (**F**) Quantification of PH3 staining; each cyst containing PH3^+^ spermatogonia was scored as one mitotic event (mean ± s.e.m. of three independent experiments, N testes/genotype). NS, not significant (*p* = 0.28) by two-tailed unpaired Student’s t-test. (**G**) Quantification of germ cell size in wild-type (*wt*) and *p53*^*-/-*^ (*p53*^*E8*^) adult *Drosophila* testes, 4 days post-eclosion (mean ± s.d., N = 4 individuals/genotype). (**H**, **I**) Bam immunostaining of *wt* (**H**) and *p53*^*-/-*^ (*p53*^*E8*^, **I**) adult *Drosophila* testes. The hub region is indicated by a white asterisk. Nuclei are stained with DAPI. Scale bar, 20 μm.

**Fig 4:**
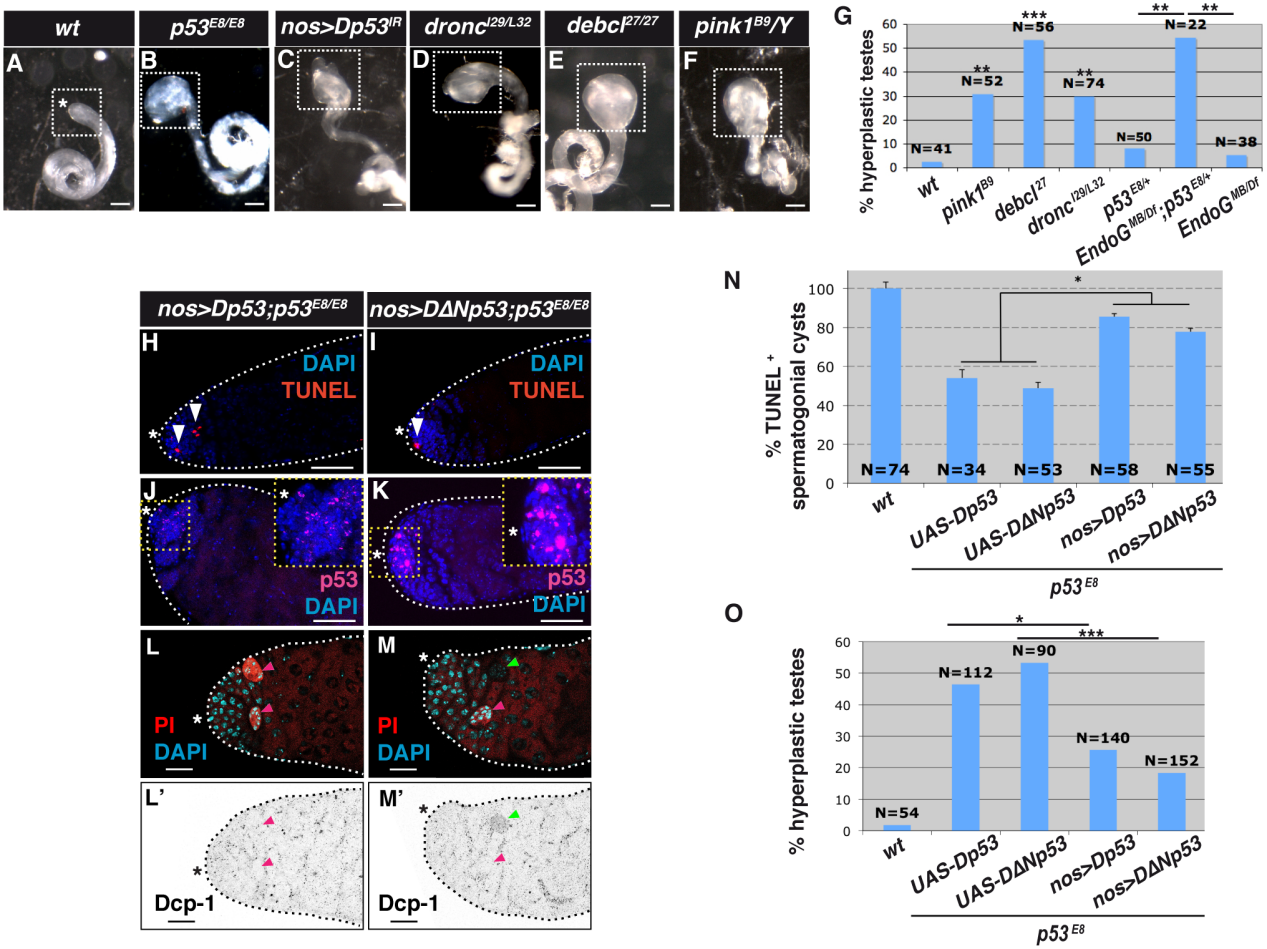
p53-dependent necrotic cell death suppresses testicular hyperplasia in *Drosophila*. (**A**-**F**) Bright field images of testes from adult wild-type (*wt; w*^*1118*^, **A**) flies and flies lacking genes required for germ cell death (**B-F**). The testis apical tip and hub region are indicated by the white dotted square and white asterisk, respectively. Scale bar, 100 μm. (**G**) Frequency of testes with apical tip hyperplasia (mean ± s.e.m. of three independent experiments, N testes/genotype). **p < 0.01, ***p < 0.001 versus *wt* flies by Fisher’s exact test. (**H**-**K**) TUNEL staining (**H**, **I**) and p53 immunostaining (**J**, **K**) of spermatogonial cysts (white arrowheads in **H**, **I**) in adult testes from *p53* mutant flies expressing *Dp53* (**H**, **J**) and *DΔNp53* (**I**, **K**) under the control of the *nanos [nos]-gal4* driver. Nuclei are stained with DAPI and the hub region is indicated by the white asterisk. Scale bar, 40 μm. (**L**-**M'**) Propidium iodide (PI) staining (**L**, **M**) and cleaved Dcp-1 immunostaining (**L’**-**M’**) of *p53* mutant fly testes expressing *Dp53* (**L**-**L’**) and *DΔNp53* (**M**-**M'**) under the control of the *nanos [nos]-gal4* driver. PI^+^ and cleaved Dcp-1^+^ spermatogonial cysts are indicated by magenta and green arrowheads, respectively. Nuclei are stained with DAPI and the hub region is indicated by a white (**L**, **M**) or black (**L'**-**M'**) asterisk. Scale bar, 20 μm. (**N**) Quantification of TUNEL^+^ spermatogonial cysts expressed as % of *wt* (mean ± s.e.m. of three independent experiments, N testes/genotype). *p < 0.05 by two-tailed unpaired Student’s t-test. (**O**) Frequency of adult testes with apical tip hyperplasia in flies of the indicated genotypes (mean ± s.e.m. of three independent experiments, N testes/genotype). *p < 0.05, ***p < 0.001 by Fisher’s exact test.

To assess the physiological role of p53-dependent necrosis, we examined the morphology of testes from *wt Drosophila*, flies carrying a *p53*^*-/-*^ null mutation, or flies with spermatogonia-specific silencing of *Dp53/TAp53*. Compared with *wt* flies, flies with whole-body or spermatogonia-specific loss of *p53* had testes displaying apical tip hyperplasia (Fig 3A–3C), ranging from mild (intumescence) to severe (massive outgrowth) based on morphology and size (S2A and S2B Fig). Hyperplasia is a typical precancerous condition resulting from the accumulation of cells that divide abnormally and/or escape cell death [34, 35]. Hyperplasia due to excessive proliferation is typically observed in flies lacking Bam, a factor required for maturation of spermatogonia (Fig 1A), in which the entire testes are filled with undifferentiated and stem-like GCs [36]. However, we observed no significant difference in the expression level of the proliferation marker PH3 between *wt* and *p53*^-/-^ testes (Fig 3D–3F), indicating that hyperplasia in *p53*^*-/-*^ flies was not due to uncontrolled proliferation. Nevertheless, we cannot exclude the possibility that small variations in cell cycle duration could affect the degree of hyperplasia in *p53*^-/-^ testes. Importantly, we saw no significant difference in the sizes of differentiating spermatogonia and spermatocytes in *wt* and *p53*^-/-^ testes (Fig 3G), indicating that hyperplasia in *p53*^*-/-*^ flies was not due to the expansion of individual cells. Rather, we observed an accumulation of maturing spermatogonia (Fig 3H and 3I) positive for the spermatogonial marker Bam (Fig 1A) as well as spermatocytes in *p53*^*-/-*^ testes (S3A and S3B Fig). Staining for the membrane marker aPKC showed that the accumulated cells were grouped in irregularly shaped cysts, and aberrant spermatid morphology and location were observed throughout the testes (Fig 3A–3B’ and S3A and S3B Fig). These findings suggest that testicular hyperplasia in *p53*^*-/-*^ flies results from a failure of GCs to undergo cell death, allowing them to differentiate and accumulate at the apical tip, rather than from excessive proliferation or growth. To determine whether the hyperplasia resulted from reduced necrosis of spermatogonia, we examined the testes of a number of flies defective in GC death due to loss-of-function mutations in *Dronc*/*Casp-9*, the mitochondrial factors *Debcl/Bax*, *Pink1*, *Drosophila Endonuclease-G* (*EndoG*), and *Drosophila Omi/HtrA2*, and the lysosomal factors *Drosophila DNAse II*, *Drosophila Cathepsin-D*, *Dor* (fly homolog of *VPS33A*), and *Car* (fly homolog of *VPS18*). We found that the *Dronc/Casp-9* mutant and all of the mitochondrial mutants tested except *Omi* exhibited testicular hyperplasia (Fig 4A–4G and S2B and S2C Fig). In the case of *EndoG* mutant, the hyperplastic phenotype was revealed by genetic interaction experiments with *p53* mutation (*p53*^*E8/+*^ background) (Fig 4G and S2C Fig). None of the lysosomal genes tested exhibited an hyperplastic phenotype to the exception of *Cathepsin-D* mutation, which induced hyperplasia when combined with the *p53* mutation. It may be because the lysosomal effectors act downstream of the mitochondria, and alternative executioners of cell death may be activated under these conditions. Collectively, it suggests that *EndoG* and *Cathepsin-D* act in the same or parallel pathways than *p53* for the execution of necrosis. Notably, expression of *Dp53/TAp53* or *DΔNp53/TAΔNp53* specifically in *p53*^-/-^ spermatogonia restored PI and TUNEL staining and inhibited apical tip hyperplasia (Fig 4H–4O). Interestingly, while no activated caspase (Dcp-1) expression was observed in spermatogonia re-expressing *Dp53/TAp53*, occasional positively stained spermatogonia were seen upon *DΔNp53/TAΔNp53* re-expression (Fig 4L–4M’). Collectively, these results indicate that re-expressed Dp53/TAp53 and DΔNp53/TAΔNp53 isoforms serve redundant tumor-suppressive functions and reduce tissue hyperplasia during spermatogenesis. The data also show that Dp53/TAp53 suppresses hyperplasia by inducing necrosis, whereas DΔNp53/TAΔNp53 is able to induce both necrosis and apoptosis. Similarly, re-expression of *Dronc*^*WT*^, *Dronc*^*C>A*^, *ΔNdronc*, *or ΔNdronc*^*C>A*^ was sufficient to restore GC death and to suppress hyperplasia in *Dronc*/*Casp-9* mutant testes (Fig 2B–2I and S4A and S4B Fig).

The p53 family member p73 has a key role in sperm differentiation and maturation but not in cell death in mouse spermatogenesis [37, 38]. Thus, we asked whether p53, which is the sole p53 family member in the fly, regulates sperm differentiation in *Drosophila*. For this, we compared expression of early and late spermatogenesis markers in *wt* and *p53*^*-/-*^ *Drosophila* testes. Early differentiation steps in *p53*^*-/-*^ testes appeared normal, as shown by expression of the somatic hub cell marker Fasciclin III, the GC marker Vasa, and the spermatogonia marker Bam (Figs 1A, 1F, 1G, 3H, 3I and S3A and S3B Fig). These data are consistent with a previous analysis of *p53*-mutant testes [26] and indicate that *p53*^*-/-*^ spermatogonia and spermatocytes undergo normal differentiation. We also detected cysts containing 64 spermatids, confirming that *p53*^*-/-*^ spermatocytes underwent meiosis (S3F Fig). Some onion-stage nebenkern spermatids showed micronuclei or had no apparent nuclei, however, suggesting a defect in chromosome segregation [39] (S3D Fig). Finally, we examined the effect of p53 loss on spermatid individualization, a process that is dependent on caspase catalytic activity and is critical for sperm maturation (Fig 1A). Sperm individualization requires non-apoptotic functions of several apoptotic factors, including *Dark/Apaf-1*, *Dronc/Casp-9*, and *Dredd (*fly homolog of *caspase-8*), as well as effector caspases, to progressively degrade cytoplasmic substrates in migrating cystic bulges [14, 40] (Fig 1A). We observed activated Caspase-3 staining in *p53*^*-/-*^ elongated sperm similar to that of *wt* flies (S3I–S3I” and S3J–S3J” Fig), but cystic bulges were rarer and the few migrating individualization complexes (IC) were often morphologically abnormal in the mutant (Fig 1A and S3L Fig). This phenotype is reminiscent of *Dronc*/C*asp-9* mutant testes in which active Drice/Casp-3 is present in elongated cysts, but cystic bulges and waste bags are largely absent and actin investment cones in the IC are uncoordinated [14] (Fig 2K). These results suggest that p53 may regulate sperm individualization in addition to its function in the regulation of necrosis in spermatogonia.

We next investigated whether the observed individualization defects in *p53* or *Dronc/Casp-9* mutant testes could lead to hyperplasia. We examined flies mutant for apoptotic genes whose loss of function is known to cause individualization defects, including *Dark/Apaf-1*, *Cyt-c-d* (fly homolog of *Cyt-c*), *Dredd/Casp-8*, and *Dcp-1*, as well as flies expressing the caspase inhibitors *p35* or *Diap1* (the main *Drosophila* IAP [Inhibitor of Apoptosis] gene). We found that none of these flies exhibited testicular hyperplasia (S5 Fig). Together with the observation that re-expression of *Dronc*^*C>A*^ under the control of endogenous promoter sequences was sufficient to suppress hyperplasia but not individualization defects in *Dronc*/*Casp-9* mutant testes (Fig 2J–2L and S4A and S4B Fig), these results indicate that testicular hyperplasia is not due to defects in sperm individualization but to the inhibition of programmed necrosis in *p53* and *Dronc/Casp-9* mutants [33].

### p53 is required for heat-induced necrosis during mouse spermatogenesis

**Fig 5:**
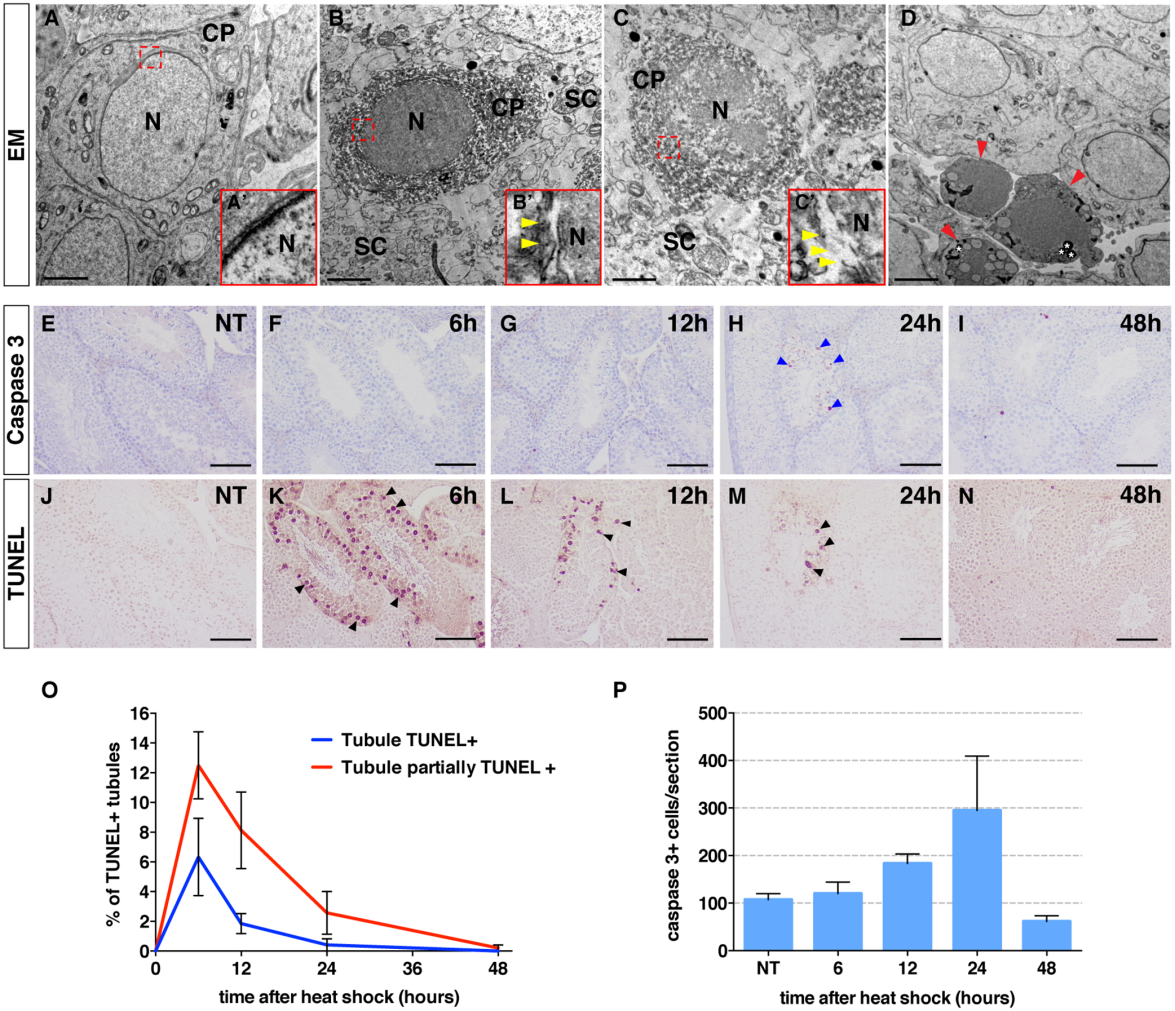
Mild hyperthermia induces germ cell necrosis during mouse spermatogenesis. (**A**-**D**) Electron micrographs of non-treated (**A**) or heat-shocked mice testes at 6 (**B**-**C'**) and 24 hours (**D**) after heat shock, showing cellular morphological hallmarks of necrosis (**B**-**C'**) or apoptosis (**D**, red arrowheads). White asterisks indicate extranuclear densities observed in the cytoplasm that may reflect an unrelated acid phosphatase activity previously observed in GCs (**D** [67]). Insets (red squares in **A**-**C'**) show ruptured nuclear membranes (yellow arrowheads in **B'** and **C'**). N and CP indicate nucleus and cytoplasm, respectively. SC indicates Sertoli cells. (**E**-**I**) Sections of non-treated (NT, **E**) or heat-shocked testes from 6–8-week-old wild-type mice stained with anti-cleaved caspase-3 antibody (**E**-**I**) at the indicated time after heat-shock. Blue arrowheads (**H**) indicate cleaved caspase-3^+^ cells. Scale bar, 100 μm. (**J**-**N**) Sections of non-treated (NT, **J**) or heat-shocked testes from 6–8-week-old wild-type mice stained with TUNEL (**J**-**N**) at the indicated time after heat-shock. Black arrowheads (**K**-**M**) indicate TUNEL^+^ cells. Scale bar, 100 μm (**J-N)**. (**O**) Quantification of the total fraction of tubules containing TUNEL^+^ cells (blue curve) and the fraction of tubules partially filled with TUNEL^+^ cells (red curve) at the indicated time after heat shock. (**P**) Quantification of activated caspase-3^+^ cells per section (mean ± s.e.m) at the indicated times after heat shock.

The dual role of *Drosophila* p53 in GC death and spermatogenesis is recapitulated by p53 family proteins in mammals where TAp53 and TAp73 have been shown to regulate spermatogonial apoptosis and spermiogenesis, respectively [16, 37, 38]. To determine whether p53 has retained the ability to regulate GC necrosis in mice, we first looked for spontaneous GC death at sexual maturity in the testes of wild-type and *p53*^*-/-*^ mice. Testes of *p53*^*-/-*^ mutant mice appeared morphologically normal with seminiferous tubules containing mature spermatozoa anchored in the central lumens (S6A–S6B” Fig). This is consistent with previous publications showing that *p53*^*-/-*^ mutant mice are fertile [37, 41]. TUNEL^+^ cells were rare in both wild-type and *p53*^*-/-*^ mutant mice, suggesting that spontaneous GC death takes place very sporadically at this age. Therefore, we used a mild heat shock to induce a burst of cell death in 6–8-week-old wild-type mice [42]. We observed a rapid increase in the number of GCs showing hallmarks of necrosis, including the characteristic nuclear membrane rupture, granulated cytoplasm, and absence of cell shrinking (Fig 5A–5C’ and S6C and S6D Fig), with a maximum level after 6 hours and lower levels after 12 or 24 hours. This suggests that mild hyperthermia induces a rapid wave of necrosis in mouse testes, which is further supported by the lack of activated Caspase-3 induction at 6 hours (Fig 5E, 5F and 5P). TUNEL staining readily detected seminiferous tubules partially or completely filled with dying cells throughout the testes (Fig 5J–5O). At 24 hours, however, TUNEL^+^ GCs were co-stained with the activated Caspase-3 antibody. Although these dying GCs did not exhibit prominent chromatin condensation, as previously observed in apoptotic germ cells [43], they did show cytoplasmic and nuclear shrinkage reminiscent of apoptosis (Fig 5D, 5H and 5P), which is also in agreement with previous studies [42]. These results therefore indicate that mild hyperthermia induces rapid GC necrosis at 6 hours, followed by GC apoptosis at 24 hours.

**Fig 6:**
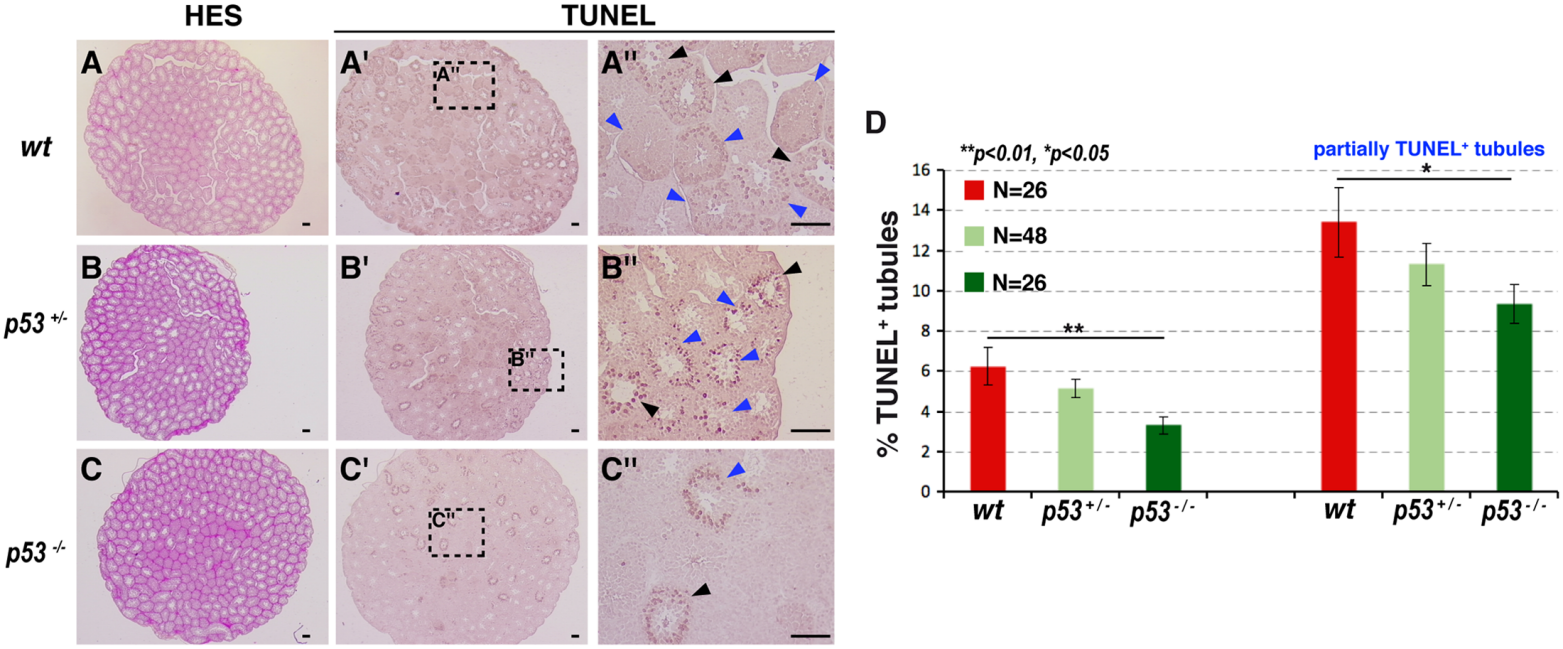
Germ cells in the testes of *p53*-deficient mice are resistant to heat-induced necrosis. (**A**-**C”**) Sections of heat-shocked testes from 6–8-week-old wild-type (*wt*, **A**-**A”**), *p53*^*+/-*^ (**B**-**B”**), and *p53*^*-/-*^ (**C**-**C”**) mice, counterstained with HES (**A**, **B**, **C**) and stained with TUNEL (**A'**, **A” B'**, **B”**, **C'**, **C”**). Black and blue arrowheads indicate seminiferous tubules fully and partially filled with TUNEL^+^ cells, respectively. Scale bars, 200 μm (**A**, **A', B**, **B', C**, **C'**) and 50 μm (**A”**, **B”**, **C”**). (**D**) Quantification of the total fraction of seminiferous tubules containing TUNEL^+^ cells (left) and the fraction of seminiferous tubules partially filled with TUNEL^+^ cells (right) shown in **A**-**C”** (mean ± s.e.m. of N testes/genotype). *p < 0.05, **p < 0.01 by Welch’s two-sample t-test.

Importantly, we observed a clear reduction in the number of TUNEL^+^ seminiferous tubules in the testes of *p53*^*-/-*^ mice compared with wild-type mice at 6 hours (Fig 6A’, 6A”, 6C’, 6C” and 6D). These data demonstrate that *p53* is required for heat-induced GC necrosis during mouse spermatogenesis and reveal a conserved function for *p53* in regulating male GC necrosis.

## Discussion

Apoptosis has long been considered the only programmed form of cell death [44]. Here, we provide cellular, genetic, and morphological evidence that programmed necrosis is required for the physiological elimination of GCs during *Drosophila* spermatogenesis. We demonstrate that GCs die by a p53-dependent mechanism that requires a catalytic-independent function of Dronc/Casp-9 and displays the hallmarks of necrosis, including loss of integrity plasma and nuclear membrane integrity, as measured by PI incorporation and supported by electron microscopy. Although we also observed nuclear condensation reminiscent of apoptosis, the conclusion that GC death involves necrosis and not apoptosis is further supported by the finding that inhibition of apoptosis does not suppress GC death [13]. Chromatin condensation has also been reported in necrosis, suggesting that this event cannot unambiguously distinguish between apoptosis and necrosis. Rather, breakdown of membrane integrity has been proposed to be the best hallmark of necrosis [45, 46]. Based on our current data and previous work, we propose that GC death should be classified as necrotic cell death.

We found that regulated GC necrosis requires the *p53* gene as well as mitochondrial and lysosomal factors, and may be an essential process for the elimination of damaged/unfit spermatogonia during mitosis. We also showed that a wave of p53-dependent necrosis can be rapidly induced by mild hyperthermia during mouse spermatogenesis, demonstrating that this mechanism of GC homeostasis has been conserved between flies and mice. To our knowledge, physiological GC death in *Drosophila* is the first paradigm of programmed necrosis resulting in cell elimination during animal development, and it provides evidence that p53-dependent necrosis is among the primordial functions of p53 [12]. Based on the importance of the non-apoptotic functions of p53, Debcl/bax, and Dronc/Casp-9 in necrotic cell death (this work and [13, 22, 47]), we speculate that necrosis may actually be the primordial function of those genes and that they later evolved to be executioners of apoptosis.

The mechanisms by which p53 activates necrosis during spermatogenesis are unclear. p53-dependent transcription was selectively observed in necrotic spermatogonia, suggesting that programmed necrosis requires the expression of as yet unknown p53 transcriptional targets [48]. Of note, p53 is functional in testicular GC tumors and renders them highly sensitive to chemotherapy [49, 50]. If testicular cancer cells are also sensitive to p53-dependent necrosis, GC tumors may be useful models for testing experimental therapies for apoptosis-resistant cancers. Intriguingly, a recent study showed that necrosis/pyroptosis mediated by Gasdermin E/DNFA5, a caspase substrate, is induced by exposure of human and mouse cancer cells to chemotherapy; however, healthy mouse tissues were also affected by chemotherapy [51]. This finding supports the notion that necrotic cell death is a physiological process that suppresses tumor formation, but also it suggests that necrosis and the associated release of intracellular content may be responsible for some of the toxicity associated with cancer therapies.

We demonstrated that a protease-independent and Dark/Apaf-1-independent function of Dronc/Casp-9 is required for the execution of necrosis during *Drosophila* spermatogenesis. Previous studies have reported non-canonical functions of Dronc/Casp-9 in other processes, such as synaptic pruning and border cell migration [52, 53]. A recent study elegantly demonstrated that Dronc/Casp-9 catalytic activity is dispensable during apoptosis-induced proliferation, a process regulated by Dronc/Casp-9 ubiquitylation, in which proliferation is non-autonomously induced by apoptotic cells [9]. However, in that case, the catalytic-independent function of Dronc/Casp-9 did require Dark/Apaf-1. In contrast, programmed necrosis in GC cells did not require either the Dronc CARD domain or Dark/Apaf-1. The lack of requirement for Dark/Apaf-1 also contrasts with the classical protease-dependent function of Dronc/Casp-9 that is required for developmental apoptosis and for non-apoptotic activation of effector caspases in sperm individualization [33, 40, 54, 55, 56].

Earlier studies also showed that Dronc-induced cell death cannot be blocked by the inhibitor of effector caspases p35, and that Dronc controls autophagic cell death in salivary glands, suggesting that Dronc/Casp-9 regulates alternative cell death pathways [33, 57]. Activation of regulated necrosis by an atypical function of Dronc/Casp-9 is reminiscent of paraptosis, a regulated form of cell death with morphological features of necrosis, which is induced via the insulin-like growth factor receptor in human cells [58]. We thus propose that Dronc/Casp-9 is a central factor in the regulation of multiple death pathways [7, 9]. Further epistatic and molecular analyses would be required to determine if p53, Dronc/Casp-9, and other genes required for GC necrosis act in the same or parallel pathways.

It is still unclear why programmed necrosis instead of apoptosis is favored in *Drosophila* spermatogenesis, but it is not due to failure to express apoptotic factors. Indeed, knockdown of Diap1 or irradiation is sufficient to trigger canonical apoptosis with effector caspase activation in spermatogonia and somatic cyst cells, respectively [13, 59]. The use of programmed necrosis for elimination of spermatogonia may be favored in the testes because a non-apoptotic function of caspases is required for sperm individualization and maturation [14, 40].

A recent study showed that starvation of *Drosophila* leads to somatic cyst cell death, which then induces the non-autonomous death of spermatogonia, a process that is important to maintain tissue homeostasis during starvation [60]. However, the physiological spermatogonial cell death described in our study cannot be rescued by expression of Diap1 in cyst cells [59], indicating that programmed necrosis is a cell autonomous process.

Our conclusion that lack of GC necrosis leads to hyperplasia of the *Drosophila* testes is supported by several lines of evidence: (1) hyperplastic testes in p53 mutants exhibit reduced GC necrosis but not excessive proliferation or growth; (2) hyperplasia in p53 mutants can be suppressed by restoring GC necrosis with re-expression of *Dp53/TAp53* or *DΔNp53/TAΔNp53*; (3) hyperplasia in *Dronc/Casp-9* mutants can be suppressed by restoring GC necrosis with re-expression of wild-type *Dronc/Casp-9* or *Dronc*^*CA*^; and (4) hyperplastic testes can be induced by suppression of necrosis but not of apoptosis. Nevertheless, we cannot exclude the possibility that mechanisms distinct from necrosis could contribute to suppression of the hyperplastic phenotype in mutants with defective necrosis, as suggested by the absence of significant hyperplasia in mutants with reduced cell death, such as *Omi/HtrA2*, *DNAseII*, and *Car*. However, we do not favor the hypothesis that hyperplasia in *p53*^*-/-*^ mutants is due to excessive proliferation that is typically observed in *Drosophila bam* mutants, in which the entire testes are filled with undifferentiated and stem-like GCs [36]. Instead, GCs proceed through differentiation in *p53*^*-/-*^ testes. In this regard, it will be important to determine if hyperplasia due to loss of necrosis could evolve to cancer in an oncogenic sensitized background. Previous work has suggested an ancient role for *Drosophila* p53 in gonadal tumor suppression by restricting stem cell growth induced by oncogenic stress or DNA stressors [11]. Thus, the dual function of *Drosophila* p53 in regulating GSC growth and necrosis may act as a double barrier to preserve genomic integrity and prevent tumorigenesis, thus safe-guarding genome transmission to the offspring.

## Materials and methods

### Fly strains

*w*^*1118*^ flies were used as wild-type controls. The following mutant and transgenic flies were used: *p53*^*5A-1-]*^ [61], *p53*^*E8*^ [62], *p53RE-GFPnls* [28], *p53*^*GD11134*^ (VDRC), *UAS-Dp53*, *UAS-DΔNp53* [30], *UAS-luc*^*IR*^ (TRiPJF01355), *UAS-dronc* lines (*UAS-dronc*^*WT*^, *UAS-dronc*^*C>A*^, *UAS-ΔNdronc*^*WT*^, *UAS-ΔNdronc*^*C>A*^, *UAS-dronc*^*CARD*^, gift from Pascal Meier, ICRF, London, UK) [33]. *UAS-dark*^*IR*^ [63], *UAS-p35* (from Hyung Don Ryoo NYU, NY), *Df(3L)H99* (from Hermann Steller, Rockefeller University, NY), *debcl*^*27*^, *dredd*^*F64*^, *cyt-c-d*^*Z21091*^, *hsp83-gal4*, *dronc*^*I29*^, *dronc*^*I24*^, *dronc*^*L32*^, *ark*^*P46*^, *ark*^*82*^, *dcp-1*^*prev*^, *strica*^*4*^, *cathD*^*1*^, *car*^*1*^, *DNaseII*^*lo*^, *EndoG*^*MB07150*^, *EndoG*^*Df(2R)BSC699*^, *omi*^*DF1*^, *nos-Gal4*, *UAS-diap1*, and *pink1*^*B9*^. Flies were maintained on standard corn/yeast medium at 25°C.

### Generation of *dronc*^*WT*^ and *dronc*^*C>A*^ mutant flies

To generate *5'3'dronc*:*dronc*^*WT*^ and *5'3'dronc*:*dronc*^*C>A*^ rescue constructs, the *dronc* 3'UTR was PCR amplified from purified *yw* genomic DNA (forward primer GAATCTCGAGTTGCCGCCACTGGACATTTTATC and reverse primer CGAATCTAGACTGCGGTTTGTTGTGAAATATC with added *Xho*I and *Xba*I sites, respectively) and subcloned into the *Xho*I and *Xba*I sites of the pattB vector. The *dronc* 5'UTR and upstream promoter region were similarly PCR amplified (forward primer GAATAGATCTCACCATTTCCTCGCCCTTGC and reverse primer GAATGAATTCTCCGGATATGGCTTCCACGC with added *Bgl*II and *Eco*RI sites, respectively) and subcloned into the *Bam*HI and *Eco*RI sites of the pattB vector containing the 3'UTR insert. The complete coding sequence of *dronc* was PCR amplified from purified genomic DNA extracted from flies carrying the *uas-dronc* transgene (a gift from Pascal Meier; forward primer GAATGAATTCATGCAGCCGCCGGAGCTCGAGA and reverse primer GAATGCGGCCGCCTATTCGTTGAAAAACCCGGGA with added *Eco*RI and *Not*I sites, respectively) and subcloned into the *Eco*RI and *Not*I sites between the 3'UTR and 5'UTR inserts. Similarly, the mutant *dronc*^*C>A*^ coding region (in which the catalytic cysteine in position 318 was replaced with alanine), was PCR amplified from plasmid DNA (a gift from Hyung Don Ryoo) and subcloned between the same regulatory regions as for wild-type *dronc*.

### Whole mount propidium iodide staining of *Drosophila* testes

Testes were dissected in Schneider’s medium supplemented with insulin, incubated with 50 μg/ml propidium iodide (Sigma) overnight, fixed for 20 min in 4% paraformaldehyde (PFA) at room temperature (RT), washed, and incubated in Vectashield-DAPI for 20 min at RT.

### Whole mount antibody staining of *Drosophila* testes

Testes were dissected in PBS at 4 days post-eclosion for all genotypes except *p53*^*5A-1-4*^ (18 days post-eclosion) and *p53*^*IR*^ (10 days post-eclosion). Samples were fixed in 4% PFA/PBS for 30 min at RT, rinsed three times in 0.1% Triton X-100/PBS (PBT0.1) for 10 min at RT, and incubated with the appropriate primary antibody in PBT0.1 for 2 h at RT. Samples were then rinsed three times in PBT0.1 for 10 min at RT, incubated with the appropriate secondary antibody in PBT0.1 for 2 h at RT, and finally rinsed three times in PBT0.1 for 10 min at RT. Samples were mounted in DAPI Vectashield medium (*AbCys*) and analyzed with a Zeiss LSM710 confocal microscope. F-actin was stained with phalloidin-rhodamine (1:300; Sigma). Primary antibodies were p53 (25F4, 1:500; Developmental Studies Hybridoma Bank [DSHB]), Vasa (rat, 1:400; DSHB), aPKC (rabbit, 1:500; Santa Cruz Biotechnology), PH3 (mouse, 1:500; Millipore), Fasciclin III (mouse, 1:500; DSHB 7G10), Bam (mouse, 1:50; DSHB), cleaved caspase-3 (rabbit, 1:1000; Cell Signaling Technology), GFP (rabbit, 1:200; Invitrogen), Dsp-1 (rabbit, 1:500; [64]), and cleaved Dcp-1 (rabbit, 1:100; Cell Signaling Technology) [59, 65]. Secondary antibodies were Alexa Fluor-conjugated and used at 1:400 dilution (Molecular Probes).

### Histology and transmission electron microscopy

Testes were dissected in 0.1 M PIPES pH 7.4 and fixed in 0.1 M PIPES pH 7.4 containing 1.5% glutaraldehyde and 1% PFA for 16 h at 4°C. After rinsing with 0.1 M PIPES pH 7.4 at RT, specimens were impregnated with 1% OsO4 in 0.1 M PIPES pH 7.4 for 30 min at RT. Tissues were then progressively dehydrated with ethanol and then propylene oxide at RT, equilibrated in 50% epoxy resin in propylene oxide for 24 h at RT, and mounted in 100% epoxy resin into silicone-embedding molds. After resin polymerization for 48 h at 60°C, samples were sectioned into 2 μm semi-thin sections, which were stained with 1% toluidine blue, and 60 nm ultrathin sections, which were stained with lead citrate and examined with a Philips CM120 transmission electron microscope operating at 80 kV.

### TUNEL staining

Testes from ice-anesthetized flies (4 days post-eclosion) were dissected in PBS, fixed in 4% PFA/PBS for 30 min at RT, rinsed three times in PBT0.1 for 10 min at RT, and stained with an In Situ Cell Death kit (Roche) according to the manufacturer’s instructions. Quantification was performed as described [13].

### Quantification of *Drosophila* testicular hyperplasia

Testes from ice-anesthetized flies were dissected in PBS at 4 days post-eclosion for all genotypes (unless indicated otherwise) and scored for hyperplasia by bright field microscopy.

### Quantification of cell size

Testes from ice-anesthetized flies (4 days post-eclosion) were dissected in PBS, fixed in 4% PFA/PBS for 30 min at RT, rinsed three times in PBS, and analyzed with a Zeiss Axiovert phase contrast microscope. Cell area was measured with Fiji software.

### Analysis of *Drosophila* testis squashes

Testes from ice-anesthetized flies (4 days post-eclosion) were squashed in PBS and analyzed with a Zeiss Axiovert phase contrast microscope.

### Mouse analysis/Ethics statement

Mice were maintained in a specific pathogen-free animal facility, AniCan, and handled in accordance with the French/Europa institutional guidelines and protocols approved by the animal care and use committee (Ethics committee of CLB, animal facility of ENS, PBES and P4 laboratory; CECCAP) agreement number 2014_CLB_023.

*p53*^*+/-*^ mice (on a C57BL/6 background [66]) were interbred to generate knockout mice. Routine mouse genotyping was performed by PCR analysis of DNA purified from fingers biopsies (Red extract kit, Sigma Aldrich). Littermate male mice were euthanized at 6–8 weeks of age. Heat shock was performed on anesthetized mice by submerging the scrotal regions in a water bath at 42°C for 30 min [42]. The testes were dissected, formalin fixed, and paraffin embedded. Tissue sections (3-μm thick) were stained with hematoxylin-eosin-saffron (HES). TUNEL and caspase-3 staining was scored in a blinded fashion on both testes of each mouse. TUNEL staining of seminiferous tubules was quantified and scored as partial or completely stained and is reported as the total number of tubules for mice of each genotype. For statistical analysis, the number of testes per genotype was 26 for p53^+/+^, 48 for p53 ^+/-^, and 26 for p53^-/-^.

## Supporting information

S1 Fig Dying germ cells in the *Drosophila* testes are TUNEL^+^ and PI^+^ but not cleaved Dcp-1^+^. (**A-B’’’**) DIC analysis (**A, B**), PI staining (**A', B', A’’’, B’’’**), and TUNEL staining (**A”, B”, A’’’, B’’’**) in *w*ild-type fly testes. Yellow arrowheads indicate necrotic spermatogonial cysts co-labeled by PI and TUNEL (**A-A’’’**). Red and white arrowheads indicate necrotic spermatogonial cysts labeled by PI only (**B, B', B’’’**) and TUNEL only (**B, B”, B’’’**), respectively. Nuclei are stained with DAPI. The hub region is indicated by a white asterisk. Scale bar, 30 μm. (**C-C”**) TUNEL (**C, C'**) and cleaved Dcp-1 immunostaining (**C, C”**) in wild-type fly testes. Yellow arrowheads indicate necrotic cells (**C, C'**, in red) and individualizing spermatids (**C, C”**, in green). Nuclei are stained with DAPI. Scale bar, 20 μm. (**D, E**) Quantification of germ cell death in adult *Drosophila* testes. Results are expressed as % TUNEL^+^ cysts relative to wild-type (*wt*, **D**) or *nanos-gal4>UAS-luciferase*^*IR*^ (**E**) flies (mean ± s.e.m. of three independent experiments, N testes/genotype). ***p<0.001 by two-tailed unpaired Student’s t-test.
(TIFF)

S2 Fig Testis hyperplasia is induced by inhibition of p53-dependent necrosis during *Drosophila* spermatogenesis and is not affected by mutations in lysosomal effectors of germ cell death. (**A**) Bright field images of testes from wild-type (*wt*) and *p53*^*-/-*^ (*p53*^*E8/E8*^) adult flies. The dotted rectangles indicate apical tips and the hub region is indicated by a white asterisk (in *wt*). Scale bar, 50 μm. (**B**) Boxplot showing the median (central line), 25^th^ and 75^th^ percentiles (box edges), and 95^th^ percentiles (bars) of the testes size index for N testes/genotype (4 days post-eclosion). The internally normalized size index was calculated as the ratio D:d, where D is the apical tip diameter at distance d from the hub and d is the average tail diameter. ***p < 0.001 by Mann–Whitney test. (**C**) Frequency of adult testes (3 days post-eclosion, *nos>Dp53*^*IR*^ 9 days post-eclosion) with a hyperplastic apical tip (N testes/genotype). **p < 0.01, NS = not significant versus *wt* flies by Fisher’s exact test.
(TIF)

S3 Fig *Drosophila p53* mutant germ cells accumulate in the testis apical tip and proceed through mitosis and meiosis, but spermatids are partially defective. (**A, B**) aPKC (red) and Fasciclin III (green; insets) double immunostaining in wild-type (*wt*, **A**) and *p53*^*-/-*^ (*p53*^*5A-1-4*^, **B**) adult *Drosophila* testes. Nuclei are stained with DAPI. Scale bar, 40 μm. (**C, D**) Phase contrast images of squashed *wt* (**C**) and *p53*^*-/-*^ (*p53*^*E8*^, **D**) adult *Drosophila* testes. Yellow arrowheads indicate normal post-meiotic, onion-stage, round spermatids containing nuclei (white dots) adjacent to characteristic Nebenkern mitochondria derivatives (black dots) in a 1:1 ratio. Magenta arrowheads in **D** indicate onion-stage spermatids with micronuclei or undetectable nuclei. Scale bar, 10 μm. (**E-H**), Electron micrographs of *wt* (**E, G**) and *p53*^*-/-*^ (*p53*^*E8*^, **F, H**) adult *Drosophila* testes. Post-meiotic 64-spermatid cysts are marked by white dashed ovals in **E** and **F**. Individualizing spermatids in (**G, H**), each containing one axoneme (labeled *a*), one major (*M*) and one minor (*m*) mitochondrial derivative, appear disorganized in *p53*^*-/-*^ testes (**H**). Scale bars, 2 μm (**E, F**) and 200 nm (**G, H**). (**I-J”**) Cleaved caspase-3 immunostaining in *wt* (**I, I', I”**) and *p53*^*-/-*^ (*p53*^*5A-1-4*^, **J, J', J”**) adult *Drosophila* testes. The hub region is indicated by a white asterisk (**I, I', J, J'**), waste bags by arrows (**I, I', J, J'**), and cystic bulges by arrowheads (**I, I”, J, J”**). Scale bar, 40 μm. (**K, L**) Phalloidin staining of F-actin-rich investment cones (arrowheads and insets) in *wt* (**K**) and *p53*^*-/-*^ (*p53*^*E8*^, **I**) adult *Drosophila* testes. Scale bar, 40 μm.
(TIFF)

S4 Fig Atypical Dronc function suppresses hyperplasia in *Dronc* mutants. (**A**) Frequency of adult testes with apical tip hyperplasia in *dronc*^*I29/L32*^ mutant flies expressing wild-type (*dronc*^*WT*^) or catalytically inactive (*dronc*^*C>A*^) *dronc* under the control of the endogenous promoter sequences (mean ± s.e.m. of three independent experiments, N testes/genotype). **p < 0.01 versus *wt* flies by Fisher’s exact test. (**B**) Frequency of adult testes with an apical tip hyperplasia in *dronc*^*I29/L32*^ mutant flies expressing full-length *dronc*^*WT*^, full-length catalytically inactive *dronc*^*C>A*^, CARD prodomain-deleted wild-type *dronc* (*ΔNdronc*^*WT*^), CARD prodomain-deleted catalytically inactive *dronc* (*ΔNdronc*^*C>A*^), or the CARD prodomain only (*dronc*^*CARD*^) under the control of the *nos* driver (mean ± s.e.m. of three independent experiments, N testes/genotype). **p < 0.01 versus *wt* flies by Fisher’s exact test.
(TIFF)

S5 Fig Inhibition of apoptosis does not induce hyperplasia during *Drosophila* spermatogenesis. Frequency of testes with hyperplastic apical tip in adult wild-type (*wt*) and *p53*^*-/-*^ (*p53*^*5A-1-4*^) flies, or in flies of the indicated genotypes defective for the apoptotic pathway (means of N testes/genotype).
(TIFF)

S6 Fig Absence of spontaneous germ cell death in testes of wild-type and *p53*^*-/-*^ adult mice. (**A**, **B”**) Sections of testes from 8-week-old wild-type (wt, **A, A'**, **A”**) or *p53*^*-/-*^ (**B, B'**, **B”**) mice counterstained with HES (**A**, **B**), and stained with TUNEL (**A'**, **B'**, **A”**, **B”**). Scale bars, 200 μm (**A**, **A'**, **B**, **B'**) and 50 μm (**A”**, **B”**). (**C**, **D**) Electron micrographs of non-treated (**C**) or heat-shocked mice testes at 6 hours after heat shock show normal (**C**) and necrotic (**D**) cells surrounded by Sertoli cells (SC). Red arrowheads indicate tight junctions. Nucleus (N) and cytoplasm (CP) are indicated. Scale bars, 2 μm.
(TIFF)
