# Supplementary figures and images for: p53-dependent programmed necrosis controls germ cell homeostasis during spermatogenesis
Source: PLoS Genet. 2017 Sep 25;13(9):e1007024. doi: 10.1371/journal.pgen.1007024 (PMC5629030; doi:10.1371/journal.pgen.1007024)

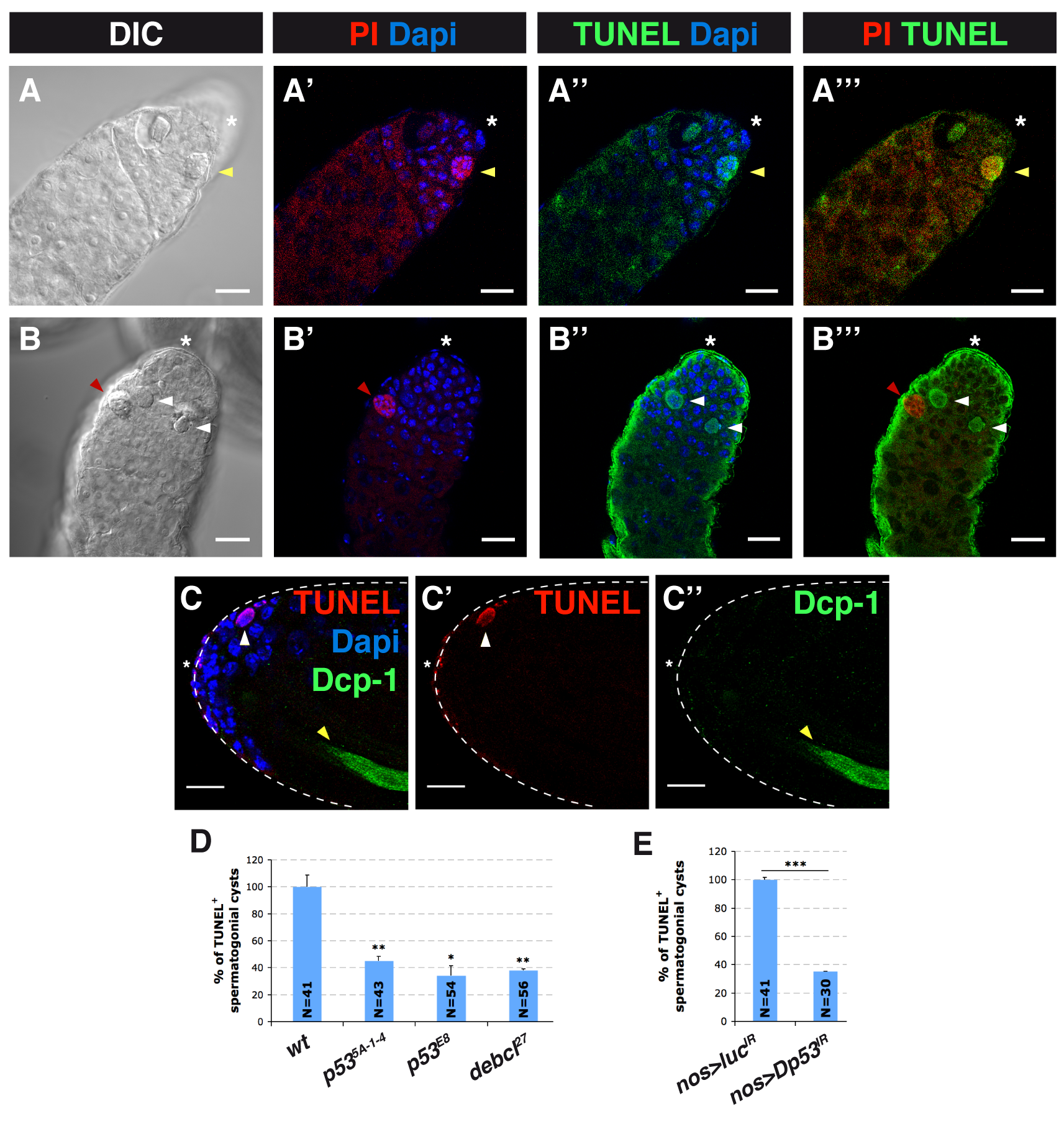

Supplement: S1 Fig — (A-B’’’) DIC analysis (A, B), PI staining (A', B', A’’’, B’’’), and TUNEL staining (A”, B”, A’’’, B’’’) in wild-type fly testes. Yellow arrowheads indicate necrotic spermatogonial cysts co-labeled by PI and TUNEL (A-A’’’). Red and white arrowheads indicate necrotic spermatogonial cysts labeled by PI only (B, B', B’’’) and TUNEL only (B, B”, B’’’), respectively. Nuclei are stained with DAPI. The hub region is indicated by a white asterisk. Scale bar, 30 μm. (C-C”) TUNEL (C, C') and cleaved Dcp-1 immunostaining (C, C”) in wild-type fly testes. Yellow arrowheads indicate necrotic cells (C, C', in red) and individualizing spermatids (C, C”, in green). Nuclei are stained with DAPI. Scale bar, 20 μm. (D, E) Quantification of germ cell death in adult Drosophila testes. Results are expressed as % TUNEL+ cysts relative to wild-type (wt, D) or nanos-gal4>UAS-luciferaseIR (E) flies (mean ± s.e.m. of three independent experiments, N testes/genotype). ***p<0.001 by two-tailed unpaired Student’s t-test. (TIFF) [file pgen.1007024.s001.tiff]

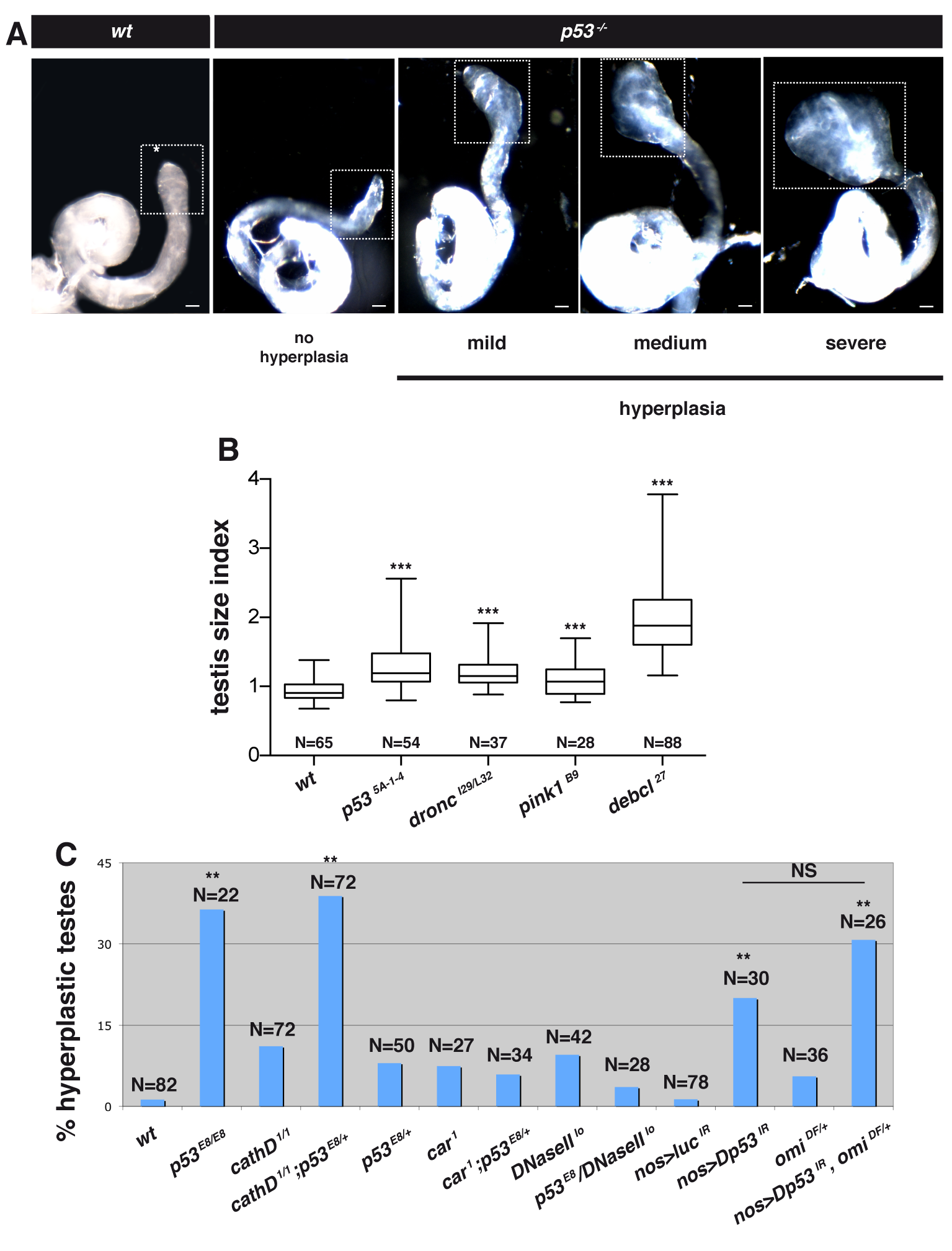

Supplement: S2 Fig — (A) Bright field images of testes from wild-type (wt) and p53-/- (p53E8/E8) adult flies. The dotted rectangles indicate apical tips and the hub region is indicated by a white asterisk (in wt). Scale bar, 50 μm. (B) Boxplot showing the median (central line), 25th and 75th percentiles (box edges), and 95th percentiles (bars) of the testes size index for N testes/genotype (4 days post-eclosion). The internally normalized size index was calculated as the ratio D:d, where D is the apical tip diameter at distance d from the hub and d is the average tail diameter. ***p < 0.001 by Mann–Whitney test. (C) Frequency of adult testes (3 days post-eclosion, nos>Dp53IR 9 days post-eclosion) with a hyperplastic apical tip (N testes/genotype). **p < 0.01, NS = not significant versus wt flies by Fisher’s exact test. (TIF) [file pgen.1007024.s002.tif]

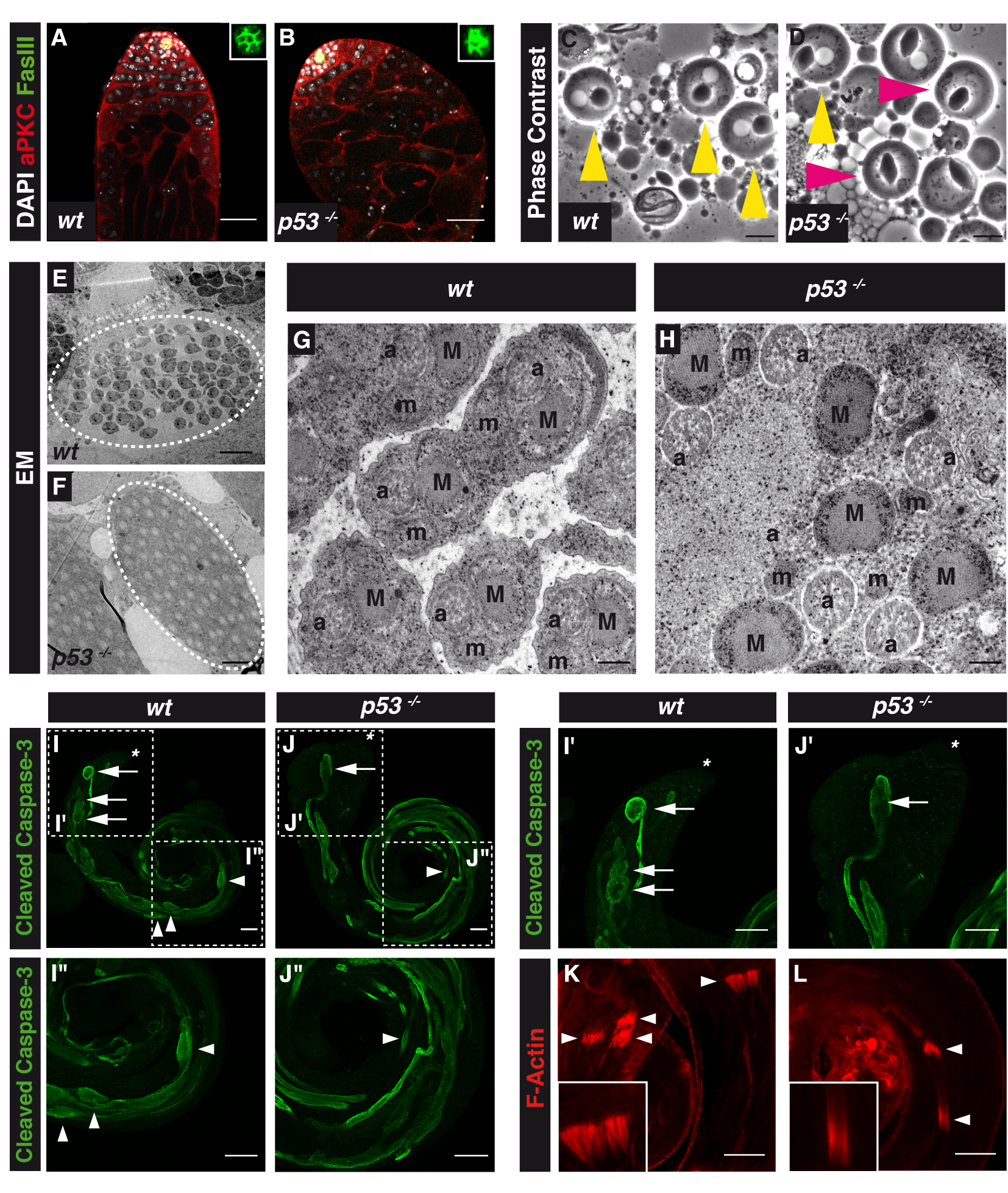

Supplement: S3 Fig — (A, B) aPKC (red) and Fasciclin III (green; insets) double immunostaining in wild-type (wt, A) and p53-/- (p535A-1-4, B) adult Drosophila testes. Nuclei are stained with DAPI. Scale bar, 40 μm. (C, D) Phase contrast images of squashed wt (C) and p53-/- (p53E8, D) adult Drosophila testes. Yellow arrowheads indicate normal post-meiotic, onion-stage, round spermatids containing nuclei (white dots) adjacent to characteristic Nebenkern mitochondria derivatives (black dots) in a 1:1 ratio. Magenta arrowheads in D indicate onion-stage spermatids with micronuclei or undetectable nuclei. Scale bar, 10 μm. (E-H), Electron micrographs of wt (E, G) and p53-/- (p53E8, F, H) adult Drosophila testes. Post-meiotic 64-spermatid cysts are marked by white dashed ovals in E and F. Individualizing spermatids in (G, H), each containing one axoneme (labeled a), one major (M) and one minor (m) mitochondrial derivative, appear disorganized in p53-/- testes (H). Scale bars, 2 μm (E, F) and 200 nm (G, H). (I-J”) Cleaved caspase-3 immunostaining in wt (I, I', I”) and p53-/- (p535A-1-4, J, J', J”) adult Drosophila testes. The hub region is indicated by a white asterisk (I, I', J, J'), waste bags by arrows (I, I', J, J'), and cystic bulges by arrowheads (I, I”, J, J”). Scale bar, 40 μm. (K, L) Phalloidin staining of F-actin-rich investment cones (arrowheads and insets) in wt (K) and p53-/- (p53E8, I) adult Drosophila testes. Scale bar, 40 μm. (TIFF) [file pgen.1007024.s003.tiff]

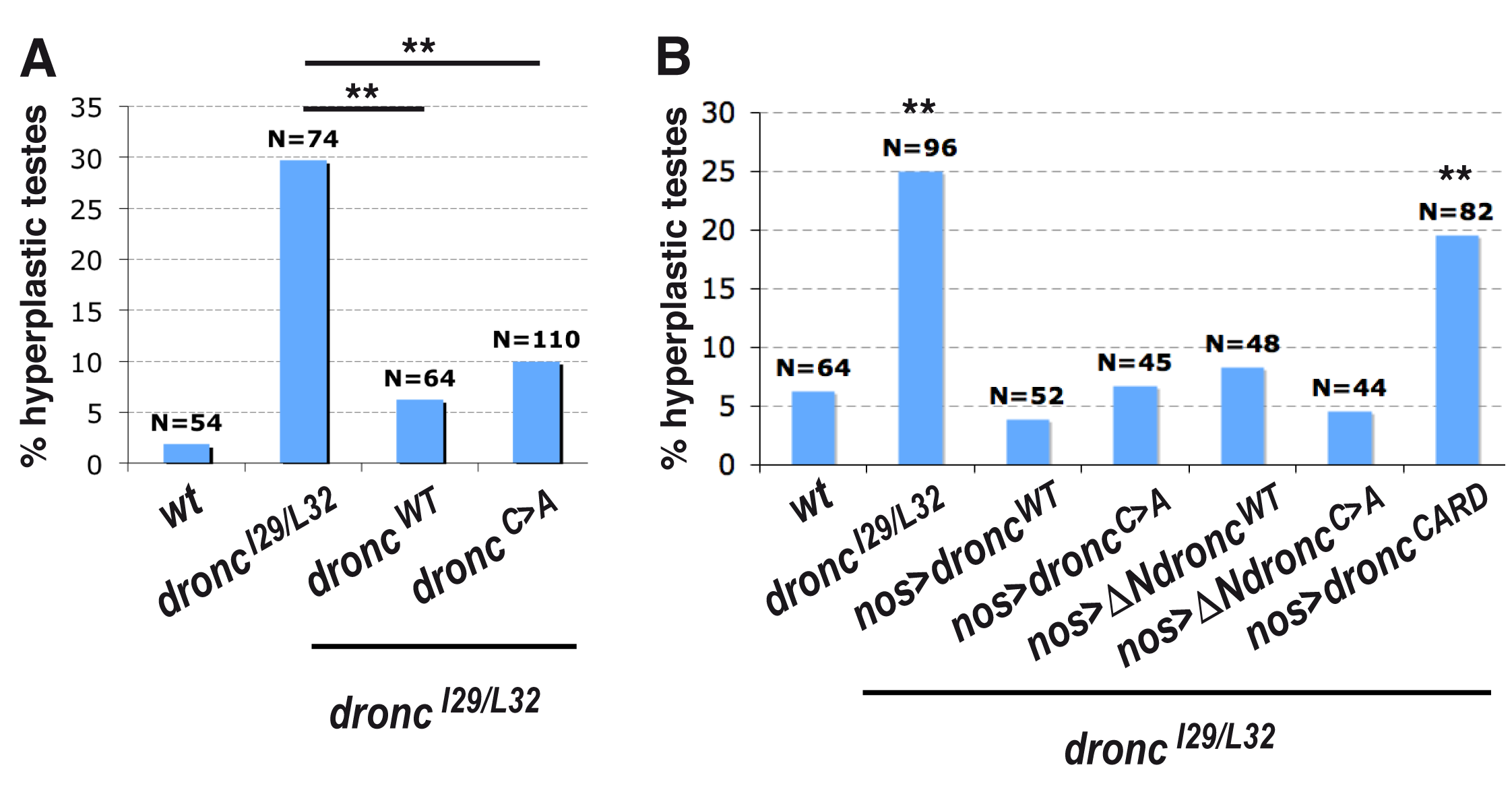

Supplement: S4 Fig — (A) Frequency of adult testes with apical tip hyperplasia in droncI29/L32 mutant flies expressing wild-type (droncWT) or catalytically inactive (droncC>A) dronc under the control of the endogenous promoter sequences (mean ± s.e.m. of three independent experiments, N testes/genotype). **p < 0.01 versus wt flies by Fisher’s exact test. (B) Frequency of adult testes with an apical tip hyperplasia in droncI29/L32 mutant flies expressing full-length droncWT, full-length catalytically inactive droncC>A, CARD prodomain-deleted wild-type dronc (ΔNdroncWT), CARD prodomain-deleted catalytically inactive dronc (ΔNdroncC>A), or the CARD prodomain only (droncCARD) under the control of the nos driver (mean ± s.e.m. of three independent experiments, N testes/genotype). **p < 0.01 versus wt flies by Fisher’s exact test. (TIFF) [file pgen.1007024.s004.tiff]

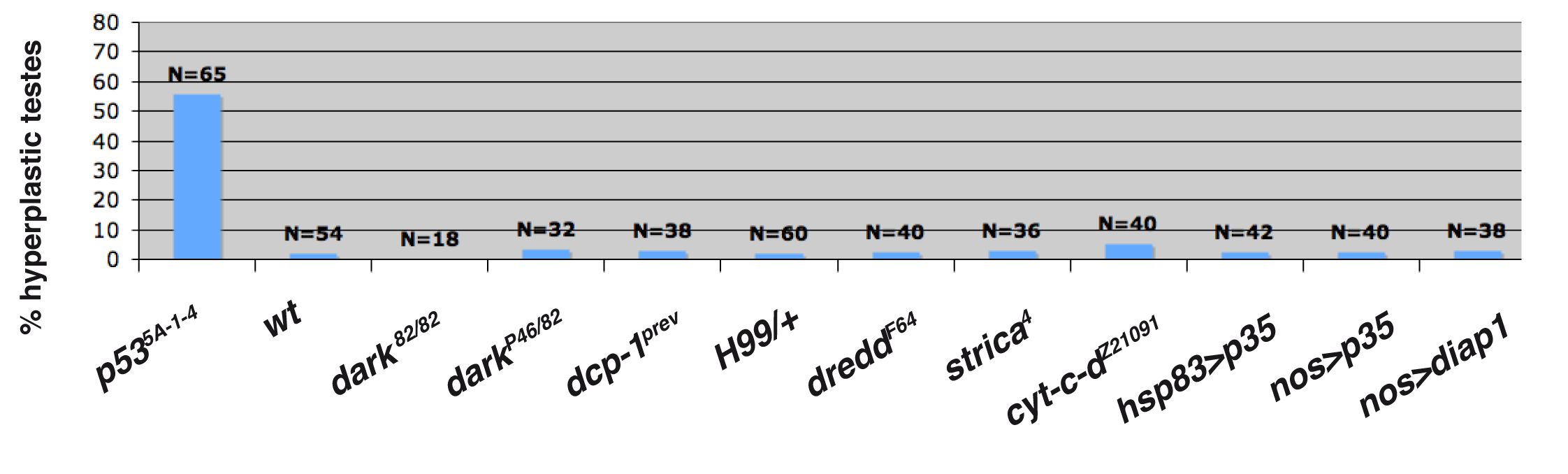

Supplement: S5 Fig — Frequency of testes with hyperplastic apical tip in adult wild-type (wt) and p53-/- (p535A-1-4) flies, or in flies of the indicated genotypes defective for the apoptotic pathway (means of N testes/genotype). (TIFF) [file pgen.1007024.s005.tiff]

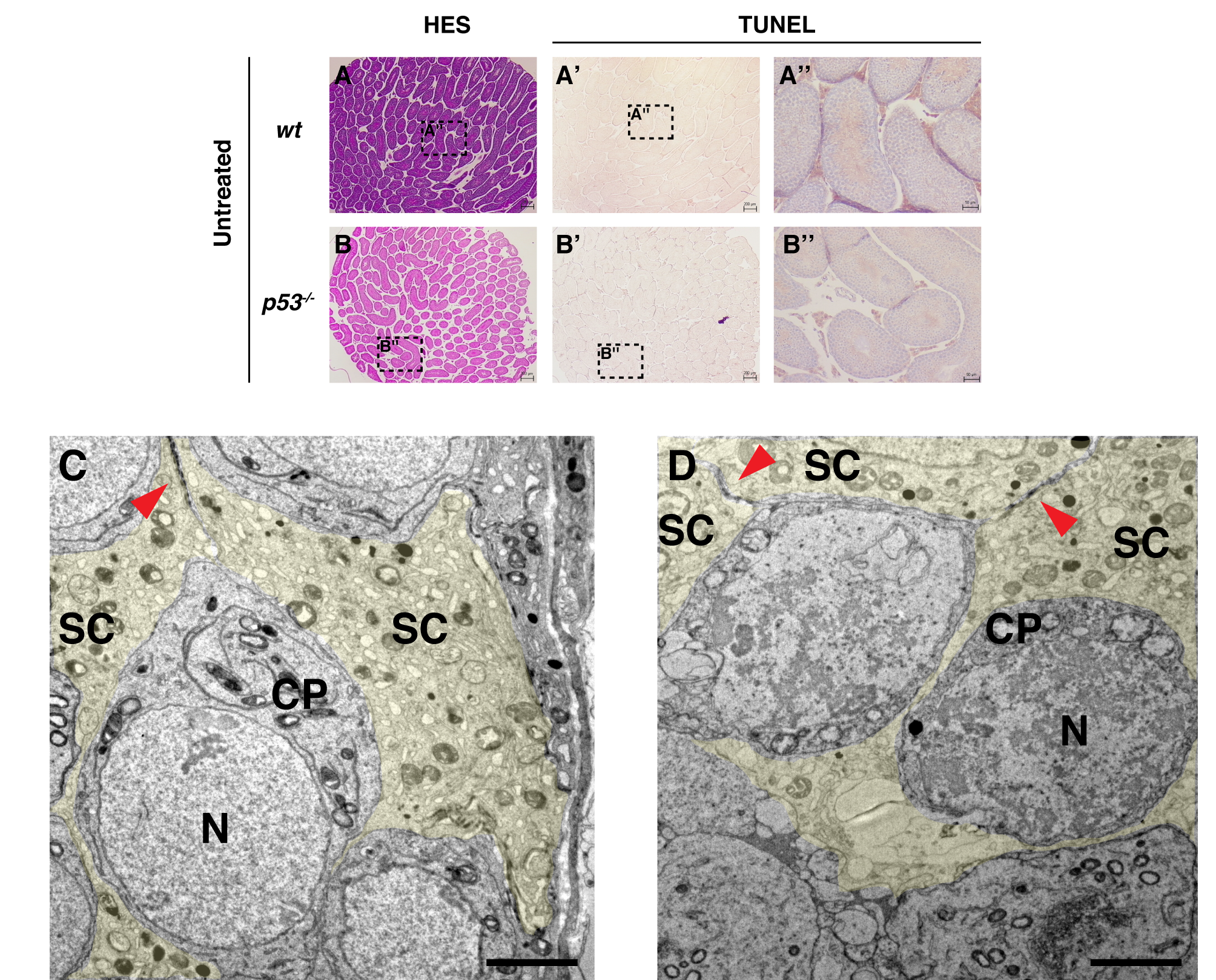

Supplement: S6 Fig — (A, B”) Sections of testes from 8-week-old wild-type (wt, A, A', A”) or p53-/- (B, B', B”) mice counterstained with HES (A, B), and stained with TUNEL (A', B', A”, B”). Scale bars, 200 μm (A, A', B, B') and 50 μm (A”, B”). (C, D) Electron micrographs of non-treated (C) or heat-shocked mice testes at 6 hours after heat shock show normal (C) and necrotic (D) cells surrounded by Sertoli cells (SC). Red arrowheads indicate tight junctions. Nucleus (N) and cytoplasm (CP) are indicated. Scale bars, 2 μm. (TIFF) [file pgen.1007024.s006.tiff]
